# Exploring the functional composition of the human microbiome using a hand-curated microbial trait database

**DOI:** 10.1186/s12859-021-04216-2

**Published:** 2021-06-07

**Authors:** Jake L. Weissman, Sonia Dogra, Keyan Javadi, Samantha Bolten, Rachel Flint, Cyrus Davati, Jess Beattie, Keshav Dixit, Tejasvi Peesay, Shehar Awan, Peter Thielen, Florian Breitwieser, Philip L. F. Johnson, David Karig, William F. Fagan, Sharon Bewick

**Affiliations:** 1 Department of Biology, University of Maryland - College Park, College Park, MD USA; 2 Research and Exploratory Development Department, Johns Hopkins Applied Physics Laboratory, Laurel, MD USA; 3 Bioengineering Department, Clemson University, Clemson, SC USA; 4 Biological Sciences Department, Clemson University, Clemson, SC USA

**Keywords:** Trait database, Functional community, Random forest, Phylogenetic correction

## Abstract

**Background:**

Even when microbial communities vary wildly in their taxonomic composition, their functional composition is often surprisingly stable. This suggests that a functional perspective could provide much deeper insight into the principles governing microbiome assembly. Much work to date analyzing the functional composition of microbial communities, however, relies heavily on inference from genomic features. Unfortunately, output from these methods can be hard to interpret and often suffers from relatively high error rates.

**Results:**

We built and analyzed a domain-specific microbial trait database from known microbe-trait pairs recorded in the literature to better understand the functional composition of the human microbiome. Using a combination of phylogentically conscious machine learning tools and a network science approach, we were able to link particular traits to areas of the human body, discover traits that determine the range of body areas a microbe can inhabit, and uncover drivers of metabolic breadth.

**Conclusions:**

Domain-specific trait databases are an effective compromise between noisy methods to infer complex traits from genomic data and exhaustive, expensive attempts at database curation from the literature that do not focus on any one subset of taxa. They provide an accurate account of microbial traits and, by limiting the number of taxa considered, are feasible to build within a reasonable time-frame. We present a database specific for the human microbiome, in the hopes that this will prove useful for research into the functional composition of human-associated microbial communities.

**Supplementary Information:**

The online version contains supplementary material available at 10.1186/s12859-021-04216-2.

## Background

Microbial communities serve important functional roles in systems ranging from the human body [1], to rhizospheres [2], up to entire ecosystems [3]. Common goals of microbiome research are to determine factors shaping microbial community assembly, and also how changes in the makeup of a community lead to changes in its overall behavior. Often, it is safe to assume that organisms with similar traits may fill similar roles, even if they are only distantly related. Thus, if we want to measure the relationship between composition and behavior, it makes sense to prioritize functional over taxonomic composition [4]. In fact, a number of studies have shown that, across nearly identical environments, taxonomic composition can be highly variable, while functional composition is largely constant. This suggests that most habitats are dominated by a stable, core functional community [5, 6].

Typically, functional analysis of microbial communities relies on genetic inference of microbial traits, specifically metabolic traits (e.g. [7]). Often, these inference methods suffer from high error rates [8, 9]. Additionally, for even moderately complex traits such as aerobicity, it is extremely difficult to make inferences from genomic data [10]. Obviously, hand-curated databases such as ours have the disadvantage of being labor-intensive to construct [11]. Others have attempted to get around this problem by using automated text-mining approaches that assign confidence levels to particular traits in specific microbes [12]. At least for type strains, however, functional information available in the literature is much better defined than automated text-mining databases imply [13]. Consequently, it is possible to assign traits to microbes with a quite high degree of confidence if one is willing to put in the time to curate the trait database. We take this laborious but precise approach and curate a domain-specific database for human associated microbes (Additional file 1). By limiting the scope of our database, we reduce the number of microbial species that we need to consider, allowing us to compile a reasonably large number of traits for an entire system of imminent importance.

We demonstrate the utility of our trait database with a number of analyses drawing on tools from machine learning and network science. As a first step we characterize the functional traits associated with different sites across the human body (e.g., stool, posterior fornix, buccal mucosa) and identify suites of traits that frequently co-occur across communities in those sites. We then build predictive models to associate specific traits with the number of body areas (e.g., gut, vagina, mouth) across which a species is found. Finally, we explore how metabolic diversity varies across sites, and predict the metabolic breadth of a species from its other traits. In all cases we adopt a phylogenetically conscious framework in which we correct model performance measures to account for non-independence due to shared evolutionary history [14].

## Results

### Revealing body-site versus trait associations

We used three complementary approaches to reveal associations of specific traits with specific body sites: (1) pairwise comparisons of mean trait values between body sites, (2) predictive modeling of sample source sites with random forests, and (3) network-based clustering of traits.

#### Pairwise comparisons between body sites

**Fig. 1:**
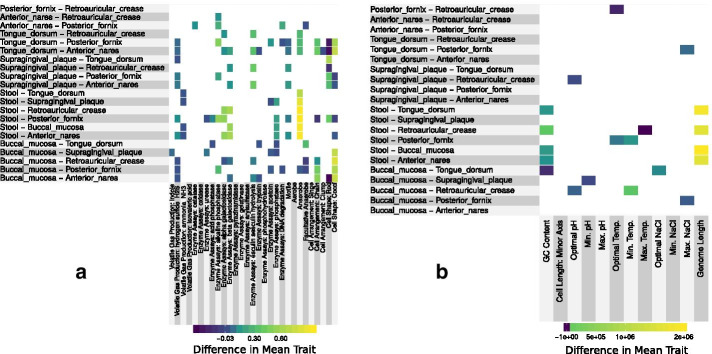
Pairwise differences in trait values between body sites (difference in means weighted by taxon abundance). Interactions that were not significant in at least two phyla are left blank. Traits separated into categories for readability: **a** qualitative with categorical values (split into dummy variables for multi-level traits) and **b** quantitative with continous values. For carbon substrate use traits see Fig. 2

**Fig. 2:**
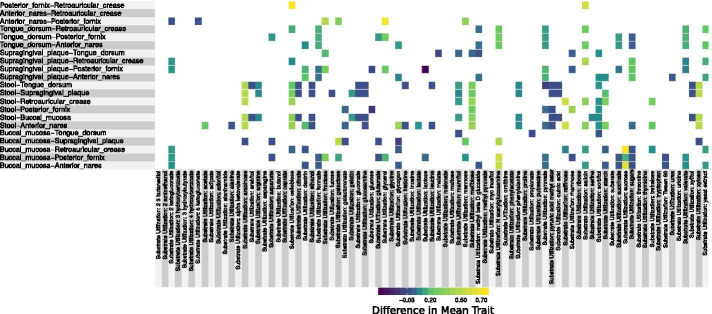
Pairwise differences in carbon substrate use frequency between body sites (difference in means weighted by taxon abundance). Interactions that were not significant in at least two phyla are left blank. Shown here are binary traits indicating the ability to grow on specific carbon sources. For other traits see Fig. 1

As seen in Figs. 1 and 2, many traits differed between body sites, even given our restriction that differences must appear across multiple phyla (see Additional file 2: S2 Fig and S3 Fig for traits with differences shown individually across all phyla, and Additional file 2: S4 Fig and S5 Fig for results on all phyla together). This is not surprising, given that different body sites provide very different environments (nutrients, temperature, oxygen, etc.) and are home to communities with very different taxonomic compositions. In keeping with pairwise results, samples clustered functionally according to body site (see Additional file 2: S1 Fig). This is similar to the results seen for taxonomic composition [15].

Some of the trends that emerged from pairwise comparisons were as expected based on knowledge of site characteristics. For example, the prevalence of anaerobes was higher in the gut (stool), a low oxygen environment, relative to other body sites. Other trends, however, reveal novel biology. Ammonia production, for instance, is under-represented in stool, while production of hydrogen sulfide gas is under-represented across the mouth (buccal mucosa, supragingival plaque and the tongue dorsum). Although there were no clear trends in carbon substrate metabolism across compound classes (e.g., alcohols, sugars), what did emerge from our carbon substrate analysis was the relative uniqueness of the different body sites in terms of resource use (see Fig. 2). This led us to build predictive models to identify those traits that most uniquely define the different locations on the human body.

#### Predictive modeling of sample source

We were able to build separate models to predict, with reasonable accuracy, if a sample came from the stool, posterior fornix, or anterior nares (Cohen’s $\kappa$ from phylogenetically-blocked cross validation: 0.436, 0.416, and 0.379 respectively; Table 1). By contrast, similar models for the mouth performed poorly ($\kappa =0.170$), and we were entirely unable to predict whether a sample came from the skin (Table 1). Difficulties with oral and skin microbiomes are likely due to the fact that trait values vary more across phyla in the mouth than at other body sites (Additional file 2: S6 Fig), and because we had very few skin samples with which to train our model (17). In fact, by restricting our analysis to only those traits that vary relatively little between phyla, we were able to increase our overall predictive ability in samples from the mouth (0.373; Additional file 2: S1 Table). To some extent, the high degree of variation in traits across phyla from the mouth probably stems from the variability in site types across the oral microbiome (tongue, plaque, etc.). However, even when we considered habitats separately, we were unable to predict whether a sample was from a specific site (tongue, plaque, or buccal mucosa), suggesting that, at least for the functions considered in our database, the functional compositions of the different oral microbial communities are similar.

**Table 1:** Cohen’s $\kappa$ for predicting sample source site

	Test				Mean
	Actinobacteria	Bacteroidetes	Firmicutes	Proteobacteria	
Stool	0.350	0.468	0.948	− 0.021	0.436
Posterior Fornix	0.340	0.351	0.560	0.413	0.416
Anterior Nares	0.430	0.579	0.268	0.240	0.379
Retroauricular Crease	0.165	0	0.033	0.038	0.059
Tongue Dorsum	0	0	0.019	0	0.005
Supragingival Plaque	0	0	0.157	0	0.039
Buccal Mucosa	0	0	0	0	0
Mouth (All)	0	0	0.677	0.004	0.170

Briefly, the trait values associated with a set of three phyla in a sample were used to train a model to predict whether a sample was from a given site on the basis of a fourth “test” phylum. Values above zero indicate predictive ability in excess of a null model accounting for the number of samples from each site

In all models, predictive ability varied across phyla. For example, while we were able to predict whether a sample came from stool based on resident Firmicutes, we were not able to do so based on resident Proteobacteria (Table 1). This may not come as a surprise, because while Proteobacteria do appear in the human gut microbiome, their abundance is typically low and their presence unreliable across individuals [16, 17]. This makes them sub-optimal predictors of sample source site.

**Fig. 3:**
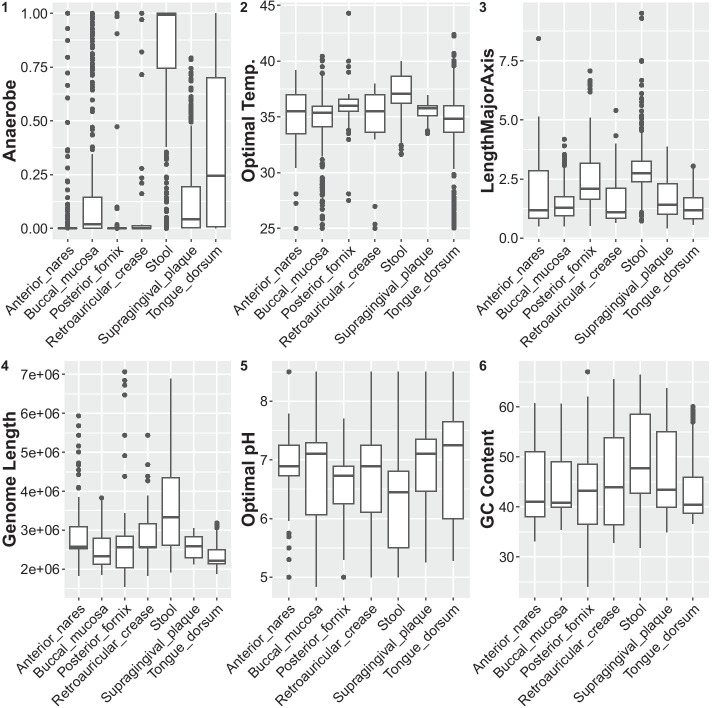
Top predictors for stool with rank shown in upper left corner. Top predictors across phyla of sample source site, for which importance scores are above the average variable importance across all predictors for all four training sets (7). Shown are mean trait values across all samples in the dataset, split up by body site. See 11, 12, and 13 for top predictors of posterior fornix, anterior nares, and mouth respectively

A number of variables were important for predicting the source site of a body sample, regardless of the set of phyla used (Additional file 2: S7 Fig, S8 Fig, S9 Fig, and S10 Fig). In Fig. 3, S11 Fig, S12 Fig, and S13 Fig we show plots of a selection of the strongest predictors across phyla for our high-performing models (stool, fornix, nares, and mouth, respectively). For example, in keeping with our pairwise analysis, the strongest predictor of a sample being from stool was a highly anaerobic resident community. Not unexpectedly, optimal temperature was also highest in stool and lowest in the mouth. Meanwhile, optimal pH was lowest in stool and highest in the mouth. Further, in keeping with the hypothesis that the gut is a complex environment, genome size was generally larger in stool and, accordingly, cell volume was also larger in this habitat. Importantly, our random forest identified traits associated with particular sites whose effects may be non-linear or context dependent (e.g., pH in the stool, formate in the anterior nares; Fig. 3, Additional file 2: S11 Fig, S12 Fig, S13 Fig). Mirroring the result in S6 Fig, the top predictors from the mouth models varied more across taxa than the top predictors for other sites (Additional file 2: S14 Fig).

#### Networks linking body sites to suites of traits

**Fig. 4:**
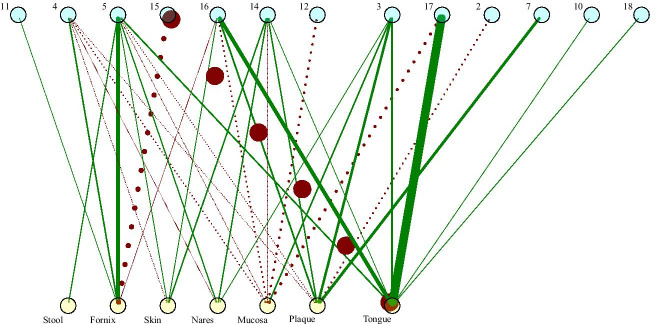
Bipartite site-cluster network, where clusters are groups of traits that frequently co-occur. Clusters are shown at the top as blue, numbered nodes. Each cluster corresponds to a group of co-occuring traits as listed in Table 2. Body sites are shown at the bottom as labeled, yellow nodes. Positive interactions (cluster common in body site) are represented by solid green lines and negative interactions (cluster uncommon in body site) are represented by dotted red lines. The strength of an interaction is represented by the with of an edge. See 16 for the same figure with positive and negative interactions separated out for ease of viewing

We inferred a network of trait associations based on the abundance of traits across samples (Additional file 2: S15 Fig). We then performed neighborhood detection to find clusters of traits that tend to covary across samples (Table 2). These clusters represent suites of traits that can be associated with a particular environment (Fig. 4, Additional file 2: S16 Fig). The combined use of butyrate and caprate, for example, are strongly negatively associated with the tongue and, to a lesser extent, the posterior fornix. Instead, the tongue is strongly associated with the combined use of adonitol and alanine. Meanwhile, the posterior fornix is associated with a complex set of traits including use of arabinose, propionate, rhamnose, succinate, and xylose, as well as production of indole and hydrogen sulfide. Interestingly, this suite of traits is positively associated with many body sites, including the nares, the tongue and stool.

**Table 2:** Inferred trait clusters with positive associations between body sites

Cluster	Traits	Positive associations
2	Use of: alaninamide, histidine, leucine, pyruvic acid methyl ester	
3	OptimalpH, Facultative, Cocci, Use of:fructose, galactose, glucose,	**Buccal mucosa**, Tongue
	lactose, mannose, methyl beta D glucoside, N acetylglucosamine, sucrose	Anterior nares, Supragingival plaque
4	LengthMajorAxis, Enzyme Assays: esculin aesculin hydrolysis,	Posterior fornix
	Use of: cellobiose, glycogen, maltose, raffinose, salicin, starch, yeast extract	
5	Max. Temp., Optimal NaCl, Min. NaCl, Max. NaCl, Genome Length, Anaerobe, Single, Clump, Rod,	**Stool**,
	Enzyme Assays: urease, acid phosphatase, alkaline phosphatase, alpha galactosidase,	**Posterior fornix**,
	beta galactosidase, acetoin, phosphatase, DNA degradation, Gas Production: indole, hydrogen sulfide,	Retroauricular crease,
	Use of: arabinose, propionate, rhamnose, succinate, Tween 80, xylose	Anterior nares, Tongue
7	Use of: phenylacetate, putrescine, quinic acid	**Supragingival plaque**
10	Use of: arginine, glycine, phenylalanine, serine, threonine	Tongue
11	Min. pH, Max. pH	Posterior fornix
12	Enzyme Assays: tellurite reductase, Use of: citrate	
14	Optimal Temp., Enzyme Assays: gelatinase, trypsin,	**Anterior nares**,
	Use of: acetate, galacturonate, glycerol, lactate,	**Retroauricular crease**,
	mannitol, melibiose, ornithine, ribose, sorbitol, trehalose	Supragingival plaque, Tongue
15	Use of: butanol, caprate	
16	GC Content, Min. Temp., Motile, Aerobe, Chain, Gas Production: ammonia, isovaleric acid,	Retroauricular crease,
	Enzyme Assays: catalase, oxidase, arylsulfatase, phosphohydrolase,	Supragingival plaque,
	Use of: aspartate, dextrin, formate, glutamate, malate, proline, pyruvate, suberate, urea, sugars	Tongue
17	Use of: adonitol, alanine	**Tongue**
18	Use of: valerate, 2 aminethanol, 2 ketogluconate, 2 3 butanediol,	Tongue
	3 hydroxybenzoate, 3 hydroxybutyrate, 4 hydroxybenzoate, 5 ketogluconate	

Bold and starred ($\star$) site names signify that a given cluster-site interaction is the strongest positive interaction observed for that site

### Generalism versus trait associations

**Fig. 5:**
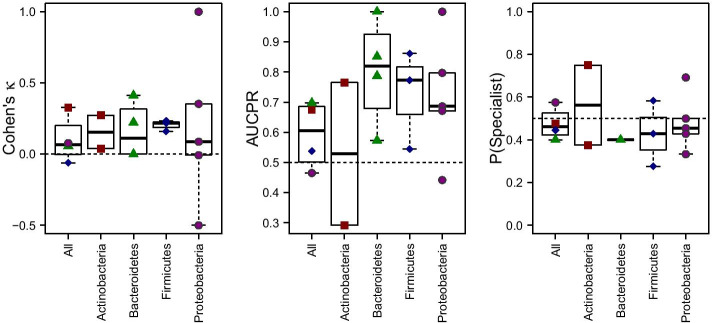
Performance of random forest models predicting generalism (binary classification, present at more than one area or not). “All” means a blocked cross-validation with each phylum as a fold (Actinobacteria: red squares, Bacteroidetes: green triangles, Firmicutes: blue diamonds, Proteobacteria: purple circles). Within each phylum we performed blocked cross-validation using classes as folds, except in the case of Bacteroidetes where all species in the dataset were in the same class and order, so that families were used as the folds. Shown are two measures of performance ($\kappa$ and area under the precision-recall curve), as well as the prevalence of specialist species in a fold ($P(\text {Specialist})$ for “probability is a specialist”)

Some human-associated microbes are found in a single body area, while others are broadly distributed across the entire human body. One hypothesis for why this might be is that there are certain traits that allow generalist species to live everywhere. To explore this possibility, we attempted to predict whether species were habitat specialists or generalists using trait data. For simplicity, we defined specialists as species that appeared in samples from only a single body area and generalists as species that appeared in samples from at least two body areas (see Methods). Specifically, we built random forest models and used blocked cross validation to obtain a phylogenetically corrected estimate of our prediction accuracy (Fig. 5). When using phylogenetically-blocked cross validation, folds correspond to clusters of related taxa (e.g., phyla, classes) rather than being chosen at random. Some phyla were more predictable than others. We predicted reasonably well whether members of Actinobacteria were generalists using the other phyla as a training set. For other phyla (Bacteroidetes, Firmicutes, and Proteobacteria), we were less successful. However, even for most of these phyla (Firmicutes and Proteobacteria), we were able to predict across Classes (see values for Cohen’s $\kappa$). Ironically, due to the relatively small number of taxa in our dataset from Actinobacteria our models performed worse when predicting within this phylum as opposed to across phyla (which requires further subdivision via cross-validation and lowers training set size).

The most important predictors varied between phyla, with little overlap (Additional file 2: S17 Fig). The exception was cell aggregation which took categorical values of chain, clump, and single, and which showed up as one of the top 5 most important predictors for three out of four phyla. Overall, however, it appears that the traits important for predicting generalism vary across phyla as a rule.

We followed up our predictive approach with individual parametric tests for phylogenetically significant trait versus generalism associations in each phylum using phylogenetic logistic regression (see Methods). Given the steep dropoff in importance after the top few predictors in Additional file 2: S17 Fig, we tested the top five most important traits for predicting whether members of each phylum were specialists, and corrected for multiple testing (Benjamini-Hochberg control of FDR, $\alpha =0.05$, *p*-cutoff = 0.021). For the Actinobacteria, the trait found to be a significant predictor of generalism was being an facultative anaerobe ($p=0.0005$, Coefficient = 1.3), whereas being an obligate anaerobe ($p=0.0002$, Coefficient = − 1.5) was associated with body site restriction. This makes sense, since facultative anaerobes are more flexible overall, and since a large number of human body sites are exposed to oxygen. For Bacteroidetes, the significant traits associated with generalism were the abilities to use yeast extract ($p=0.0023$, Coefficient = − 0.72) and aspartate ($p=0.0069$, $\text {Coefficient}=-0.55$), as well as $\beta$-galactosidase ($p=0.0101$, Coefficient = 0.73), and alkaline phosphatase ($p=0.0014$, Coefficient = 0.83) activity. Like Actinobacteria, generalist Firmcutes were also more likely to be facultative anaerobes ($p=0.0102, {\text{Coefficient}}=0.72$), while specialists were more likely to be obligate anaerobes ($p=0.0094,\,{\text{Coefficient}}=-0.75$) Other traits predictive of generalism for Firmicutes were having a small genome length ($p=0.0003,\,{\text{Coefficient}}=-1.4$), and a low GC content ($p=0.0191,\,{\text{Coefficient}}=-0.78$). For Proteobacteria the only significant trait associated with generalism was minimum growth temperature ($p=0.0197,\,{\text{Coefficient}}=0.84$).

### Metabolism

Metabolic breadth - the number of substrates used by a particular microbiome - is a measure of the diversity of functions and the flexibility of the microbial community. As such, it reflects microbiome complexity, which may, itself, be a reflection of the complexity of environmental conditions and/or resource inputs into the system. Below, we consider metabolic breadth, first across body sites, and then across microbial taxa.

#### Metabolic breadth across sites

**Fig. 6:**
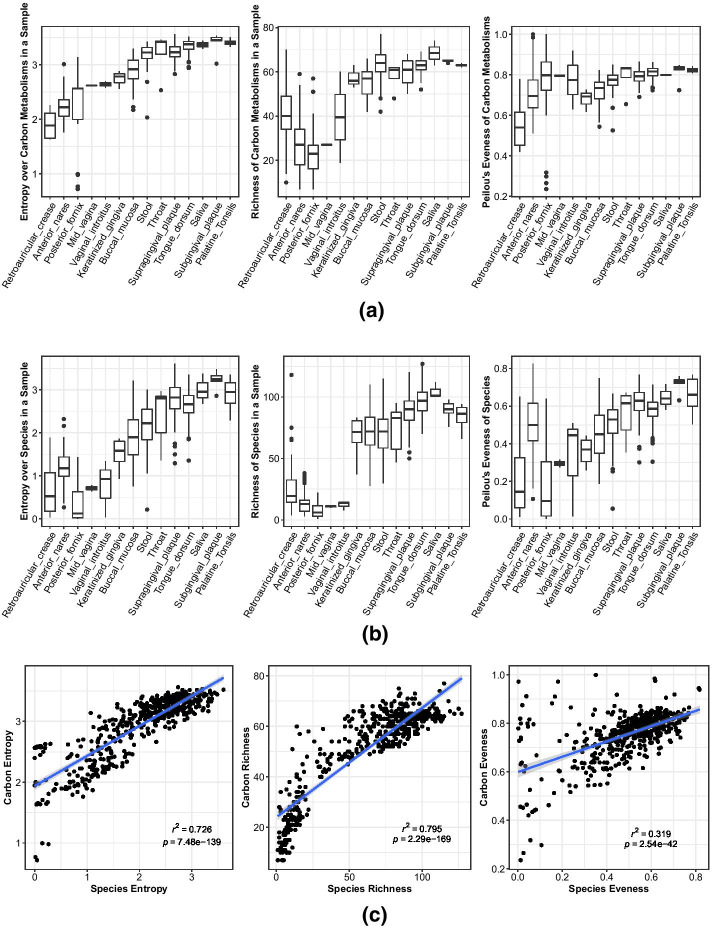
Diversity of carbon sources in a body site is largely mediated by taxonomic diversity. **a** Diversity of carbon sources used within samples. **b** Taxonomic diversity within a sample. **c** Values from (**a**, **b**) for each sample plotted against each other. Entropy (Shannon’s entropy) is a common diversity metric that integrates both the eveness and richness of items considered (carbon sources and species in panels (**a**, **b**) respectively)

Different body sites differ in the overall number of carbon substrates used by their resident microbes, with a high coverage of carbon sources in stool and the majority of oral sites and a much lower coverage of carbon sources in skin, nares and vaginal sites (Fig. 6). There are three proximate reasons why metabolic breadth could be increased in some body sites: (1) species associated with those sites may use more carbon sources on average (increased metabolic flexibility), (2) species associated with those sites may vary more among themselves in terms of which substrates they can use (increased niche differentiation), or (3) some sites may simply have a higher taxonomic diversity (increased number of niches). Although not mutually exclusive, the first mechanism suggests that carbon source availability is less predictable in time, the second mechanism suggests that strong competitive interactions structure the community, and the third suggests that there is generally a higher diversity of carbon sources available (at all times). Interestingly, we found that the high metabolic breadth observed along the alimentary tract (oral and gut sites) could be attributed almost entirely to (3) an increase in taxonomic diversity (Fig. 6) at these locations. By contrast, species from different body sites did not vary significantly in their overall metabolic capacity (Additional file 2: S18 Fig, panel a), although there were significant differences when restricting the analysis to site specialists (Additional file 2: S18 Fig, panel c). Even accounting for specialists, however, these differences did not explain overall trends in carbon source usage among sites. Likewise, while there were significant differences in the number of carbon substrates shared among species across sites, this variation did not explain overall trends in metabolic breadth (Additional file 2: S19 Fig). Indeed, in some cases, it demonstrated the opposite pattern. For example, species from skin and nares sites actually showed less overlap in substrate usage as compared to species from saliva, even though metabolic diversity in saliva was higher.

Although differences in niche differentiation did not explain body sites differences in metabolic diversity, we still found evidence of niche partitioning. In particular, diverse kinds of carbon metabolism were more evenly represented across samples than were species abundances (Fig. 6). This suggests some community-level selection to use carbon sources in a balanced way, even if the taxonomic community composition is highly skewed.

#### Metabolic breadth across taxa

Despite the fact that variation in taxon-specific metabolic flexibility did not explain trends across body sites, we still observed significant differences in the number of carbon sources used by different human-associated microorganisms. This led us to attempt to predict the number of carbon sources a species uses with moderate success. While our root mean square errors (RMSE) indicated that our models have limited predictive ability, our predicted values correlated with the actual values, suggesting that our models captured some portion of the overall trend of how substrate use varies with traits (Table 3). The only trait that was important for predicting the number of carbon substrates across all phyla was genome length (Additional file 2: S20 Fig). This is in keeping with previous findings from soil bacteria that suggest larger genome sizes are strongly correlated with more metabolic capabilities [18]. Growth temperature and pH range were important across 3/4 phyla; however, in varying forms (min., max., optimal). This may be because pH can impact the availability of carbon substrates, influencing selective pressures on utilization patterns [19]. DNA GC content was also important for 3/4 phyla (Actinobacteria, Proteobacteria and Firmicultes), which is not surprising given its relationship to genome size [20]. Finally, the presence of alpha-galactosidase activity was correlated with metabolic breadth in 3/4 (Actinobacteria, Firmicutes, and Bacteroidetes) phyla. This, too, makes sense, because alpha-galactosidases are important for breaking down and making available certain types of carbon substrates, and thus may be selected for by the same pressures that select for metabolic breadth in general.

**Table 3:** Performance of random forest models of the number of carbon substrates a species can use

Test	RMSE	*ρ*	*R*^2^ Adjusted
Actinobacteria	6.20	0.327	0.089
Bacteroidetes	6.07	0.354	0.108
Firmicutes	5.08	0.467	0.213
GeneralismSingletons Proteobacteria	8.67	0.582	0.332
Mean	6.51	0.433	0.186

## Discussion

### Traits associated with particular sites

We used three complementary approaches to demonstrate how the functional composition of the local microbial community changes across regions of the human body. First we looked at the magnitude of the difference in trait values across sites, which has the clear benefit of interpretability (Figs. 1, 2). Second, our random forest models allowed us to determine which traits were most important for predicting the body site of a sample, thus indicating which traits best discriminated between body sites (Fig. 3). Finally, our network-based approach allowed us to cluster traits into suites that frequently co-occured across samples. Clustering yielded a more comprehensive and intuitive view of the groups of traits associated with specific body sites (Fig. 4, Table 2).

Some sites are more reliably associated with traits than others. We could predict the source of a sample from the stool, posterior fornix, and anterior nares quite well based on its trait composition. On the other hand, trait values vary more across phyla in the mouth than in other body sites (Additional file 2: S6 Fig), making it difficult to predict across phyla in this body area. While a large number of candidate traits were found to be associated across sites using our three methods (see Results), several such associations were identified by multiple methods. Stool microbes prefer warmer temperatures, are anaerobic, and have large cells with large genomes. These observations are in line with our expectations based on their environment. These traits are also part of a larger suite of traits (see cluster 5 in Table 2), including the ability to use simple plant sugars (e.g. arabinose, xylose) and the production of $\alpha$- and $\beta$-galactosidase that are important for breaking down more complex galactosides into simple monosaccharides. Notably, plant and other complex carbon sources are most likely in the gut, where they are inputted as a result of host diet.

Interestingly, the strongest predictors for a sample coming from the nares and the fornix typically involved negative associations (the inability to metabolize formate or glycerol respectively, for example). This is perhaps unsurprising, since these sites are unlikely to have the rich diversity of carbon sources available in the mouth or gut. An exception is that many microbes in the nares can metabolize mannose, which is interesting given the role of mannose in the airway immune response [21, 22].

### Generalism and metabolic breadth

Generalism, it seems, is a difficult trait to predict. This may be a product of the scale at which we define generalism. First, each body site likely comprises a diversity of microenvironments (e.g. [23]), meaning that a species may move into a new site by exploiting any number of distinct niches. Second, despite the profoundly different environments provided by different body sites, they are all, nonetheless, similar by virtue of being human host-associated. Prediction of generalism across more distinct niches, for example soil versus human versus marine environments may show a broader range of generalizable traits. Despite this, for all phyla, our models had some ability to predict generalism. For the Actinobacteria we were able to predict generalism relatively well by building a model based on the other phyla. For Bacteroidetes, Firmicutes, and Proteobacteria it was difficult to generalize across phyla, but we had some predictive ability within phyla. The limited overlap in the determinants of generalism across phyla surprised us. It seems that different traits determine habitat breadth for each phylum. This is likely due to the fact that certain behaviors are more/less variable across different phyla, and thus are more/less likely to be identified as determinants of generalism overall. As an example, in phyla with a mix of aerobic, facultative and anaerobic members, oxygen use may predict generalism; however, oxygen use is unlikely to be an important predictor amongst Proteobacteria, whose members are almost never anaerobic. Lack of generality across phyla may also stem from interactions with the many microbial traits omitted from the database, which may vary systematically across phyla.

For traits identified as being associated with generalism, we confirmed links within each phylum using phylogenetic logistic regression, which assumes a parametric model of trait evolution. Aerobicity was a significant predictor of generalism in Actinobacteria and Firmicutes, with facultative organisms more likely to be generalists than anaerobes. For Firmicutes, both genome length and GC content (which are somewhat correlated [24]) were negatively associated with generalism. This is surprising, because genome length is generally positively correlated with metabolic breadth (as seen here and by others; [25]), which, in turn, is usually associated with habitat generalism. In contrast with our result, other groups have observed a positive relationship between genome length and the number of habitats in which a microbe is found in soils [18, 26]. In fact, genome size varies greatly across body sites (Fig. 1, Additional file 2: S21 Fig), with microbes in the gut having especially large genomes and those in the vagina having comparatively small ones. This suggests that the story of genome length and ubiquity is not as simple as large-genomed organisms being able to occupy more niches. Rather, different body areas appear to select for different genome sizes, likely due to the relative diversity of carbon sources available in each (Fig. 6). Being a generalist on the human body therefore appears to be more about using the handful of carbon sources that are prevalent and shared across all sites, versus using a wide range of carbon sources. This speaks to the selection pressures associated with living in host- associated niches.

As noted above, we found that metabolic breadth was related to genome length, and this relationship applied for all phyla. This is in line with the observation that the number of metabolic genes an organism has scales with its genome size [25]. We found that the overall diversity of metabolisms in a sample, though, was primarily determined by the taxonomic diversity within that sample, rather than any special feature of those species (e.g., increased metabolic breadth).

### The database approach

As with any method, our database approach suffers from several drawbacks. In exchange for the higher level of accuracy in our trait assignments, we lack the resolution of methods that predict traits based on genomic data. While our trait determinations were generally made for type strains of each species, for many traits there is a good deal of variability across strains in a species due to heterogeneous gene content [27–29]. This variability essentially adds noise to the data, meaning our analyses are somewhat less powerful than they could be with more complete information. Inference based on genomic content would not solve this problem, because these methods are, themselves, noisy [8, 9], and cannot be used to infer highly complex traits with great confidence (e.g [10]). The clearest solution, then, is to assay a large number of strains in each species for a large number of traits, though time and money of course limit pursuit of this solution.

A second drawback to our approach is that functional characterization requires a microbe to be in culture, and many species have proven resistant to culturing techniques. Historically this has been true of the many anaerobic species in the gut, but more recently high-throughput methods have been rapidly closing the gap of unculturable microbes [30, 31]. While inference methods might help temporarily fill in the gap on unculturable microbes, there is no reason to think that their state of unculturability will persist for long, suggesting that curated database approaches will be even more appealing in the future.

Third, compiling information manually is laborious and, unavoidably, leads to the introduction of occasional errors. In a previous paper [13], we used comparison to the ProTrait database [12] to determine that the error rate for our trait compilation method was $\sim 0.5\%$. This is relatively low, and comparable to the ProTrait database itself [13]. However, to improve accuracy, we encourage feedback (https://bewicklab.weebly.com/databases--packages.html). This allows for a living database that can be corrected as errors are identified and new information becomes available through culture experiments.

Finally, in most cases the largest drawback to the database approach is the absence of any universal, regularly updated trait database for microbes, though much of the required information does exist in the literature at large and some attempts have been made to capitalize on this [11, 12, 32]. Thus compilation becomes a necessary, and time-consuming step. It is our hope that our domain-specific trait database can help to alleviate this problem, at least in human microbiome research.

### Conclusions

We built and analyzed a domain-specific microbial trait database to better understand the functional composition of the human microbiome. Using a combination of phylogentically concious machine learning tools and a network science approach, we were able to link particular traits to areas of the human body, discover traits that determine the range of body areas a microbe can inhabit, and uncover drivers of metabolic breadth.

## Methods

### Input data

#### Taxon lists and taxon prevalence profiles

The HMP.ab.txt.bz2 file containing microbial compositions of human gut, vaginal, mouth, skin and oral/airway samples was downloaded from from the NIH Human Microbiome Project website (https://www.hmpdacc.org/HMSMCP/#data). From this data file, we obtained lists of microbial taxa, along with their relative abundances for each of the 690 samples from the Human Microbiome Project whole metagenome shotgun sequencing project that passed quality control [33, 34]. Using the taxa lists for each of the 690 samples, we then compiled a master list of all microbes recorded in at least one sample. This list was used to develop our trait database.

Following the convention of the Human Microbiome Project we refer broadly to body areas (gut, vagina, mouth, skin, airways) and more specifically to body sites (e.g. stool, posterior forxin, buccal mucosa, retroauricular crease, anterior nares). Each sample can be categorized as being from a specific body site within a larger body area.

#### Trait database

Using the lists of taxa generated above, we compiled a database of microbial traits. For this, we relied on Bergey’s Manual of Systematic Bacteriology [35–38] and the original journal articles describing each species. We only considered validly described species and did not include Candidatus taxa, where little information was available. In addition we ignored taxa lacking valid species descriptions. Our database contains trait information for 2260 species.

We also added entries for genus-level traits (some taxa in the analysis below could only be identified down to the genus level). For each genus, we took the value for each trait to be the consensus value across entries for species in that genus. If there was not 100% agreement among species in a genus for a particular trait, that trait value was coded as missing. Our database includes entries for 1111 genera.

In total, our database includes 155 traits. Of these 13 take on continuous values (e.g., optimal growth temperature; coded as NA when not reported), 45 are categorical (e.g., aerobe, anaerobe, faculative, etc.; coded as NA when not reported), and 97 are binary variables indicating the presence or absence of a certain activity (e.g., growth on glucose, production of hydrogen sulfide; coded as 0 when not reported or if explicitly reported as being absent).

Four phyla in particular (Actinobacteria, Bacteroidetes, Firmicutes, and Proteobacteria) had at least 50 representative taxa found in both the trait database and HMP samples. For many downstream analyses we focus on these four phyla, as they have sufficient data available to train and test phylum-specific models.

#### Mean trait value profiles

For each individual trait, we took a weighted mean of that trait’s value in a given sample using species relative abundances as weights (this amounts to averaging trait values over individuals, and the result would be identical using absolute abundances). In cases where trait values were missing for a given species, abundances were renormalized in the absence of that species, so that the weighted mean was only taken among known trait values. This yielded a matrix with traits as rows and individual samples as columns, where entries represented the mean value of a trait across microbes in a sample.

#### Phylogeny

For each taxon identified to the species level that was represented in both the trait database and HMP dataset, we downloaded a single genome from NCBI’s RefSeq database [39], with a preference for reference and representative genomes as well as completely assembled genomes. Using PhyloSift v1.0.1 [39] we identified and aligned core genes that were shared between all genomes in a phylum (phylosift search and phylosift align commands using the –besthit option). We then concatenated these alignments for phylogenetic analysis (45 genes in Actinobacteria, 48 in Bacteroidetes, 44 in Firmicutes, and 49 in Proteobacteria). Finally, we inferred a phylogeny for each phylum using RaxML v7.2.8 (options: -m GTRCAT -f a -p 456 -N autoMRE; [40]; Actinobacteria, Additional file 2: S22 Fig; Bacteroidetes, S23 Fig; Firmicutes, S24 Fig; Proteobacteria, S25 Fig).

#### Generalism

For each of the taxa represented in both the trait database and HMP dataset, we quantified the generalism of each species across the human microbiome. We calculated the number of body areas (gut, vagina, airways, skin, mouth) each taxon appeared in, requiring at least two appearances in samples from an area to be counted (331 taxa after excluding those found in only a single sample). This requirement for two appearances is intended to reduce the possibility of false-positives, where singletons are more likely to be the result of noise rather than the actual association of a taxon with a given body area. Indeed, our predictive models performed poorly when trained/tested without first filtering out singletons (Additional file 2: S26 Fig).

### Body-site versus trait associations

#### Pairwise differences in trait composition between body sites

For each trait we performed all pairwise comparisons between body sites. Statistically different mean trait values between sites were determined using a permutation test. First the mean value of each trait in each sample across individuals was calculated (see above). Then, for each trait and each pair of body sites we compared the difference in the mean trait value as our test statistic. For each pair of body sites, body site identifiers were then permuted and the mean difference in trait values was re-calculated to generate a null distribution for comparison (10,000 permutations). In order to account for multiple testing, we controlled the false discovery rate using the Benjamini-Hochberg correction, a popular method in exploratory studies ($\alpha =0.05$; [41]).

In order to control for the possibly confounding effects of phylogeny, we repeated this analysis for each phylum with a large number of species in the dataset (Actinobacteria, Bacteroidetes, Firmicutes, and Proteobacteria). Trait-comparison pairs that were found to be significant in more than one phylum were considered to be ecologically informative.

For both analyses we randomly sampled only one sample per body site for each study subject, as repeated samples from the same subject cannot be considered to be statistically independent.

#### Predicting body site from trait composition using random forests

In order to identify traits strongly associated with a particular body site we took a predictive approach that incorporated random forests for prediction with blocked cross validation [14, 42] to correct our error estimates for phylogeny. We split each sample into four individual communities for each of its constituent phyla (Actinobacteria, Bacteroidetes, Firmicutes, Proteobacteria) and calculated the mean trait values individually for each of these phyla-samples (i.e., the set of species in a sample from a given phylum). Then, leaving out the phyla-samples from one of these phyla (e.g. Actinobacteria) we fit a random forest (randomForest R package, 5000 trees, stratified sampling for uneven classes; [43, 44]) to perform binary classification on whether a phyla-sample was from a given body site (e.g. stool/not-stool) using the remaining three sets of phyla-samples. This was repeated leaving each phylum out in turn, with predictive ability calculated on the left-out phylum each time (i.e., blocked cross validation [14]). We did this for all body sites with samples from $\ge 10$ individuals available (stool, fornix, nares, retroauricular crease, plaque, buccal mucosa, tongue).

#### Trait network

Using the trait prevalence profiles we found above, we constructed trait co-occurence networks. We used the graphical Lasso method [45] to find conditionally dependent interactions between traits (tuning parameter selected using Extended Bayesian Information Criterion, EBICglasso() function in the qgraph R package; [46]).

In order to identify suites of associated traits, we then performed community detection on the resulting network using the spin-glass method (igraph R package; [47]). This approach comes from statistical physics and is based on a model used to describe particle spin states [48, 49]. There are many different graph-clustering algorithms available (and many are implemented in popular network science packages like igraph [47]), each based on different criteria and with a different tendency to either “lump” or “split” groups. The spin-glass method has the advantage of being able to account for both positive and negative interactions in the network when performing community detection, whereas most other methods ignore negative interactions. Nevertheless, a comparison with several other clustering methods (information theoretic, Additional file 2: S27 Fig; heirarchical, S28 Fig; centrality-based, S29 Fig) revealed that the methods tended to agree on group membership (with the exception of the method based on betweeness centrality S29 Fig).

We then built a bipartite network associating the trait clusters we found above with specific body sites. We performed Lasso regressions with each site as a binary outcome variable (glmnet package in R, 10-fold cross validation on mis-classification error to choose $\lambda$; [50]) and the trait values associated with that site as predictors. We then took the weight of an edge connecting each site with each cluster as the mean regression coefficient for a site with all traits in a given cluster that were retained in the model.

#### Predicting generalism using random forests

We constructed random forest models to predict generalism within each phylum (randomForest R package, 5000 trees, stratified sampling for uneven classes). For simplicity, we coded specialism/generalism as binary trait, where a species was considered a specialist if it appeared in one body area only, and a generalist if it appeared in more than one body area. Any missing trait values for a given taxon were imputed using the mean trait value in the database. The out-of-bag error estimate produced during the fitting of a random forest will give a biased estimate of model performance when observations are not independent of one another. This is potentially the case for our phylogenetically structured dataset. To get an accurate estimate of our prediction error we used blocked cross validation [14], in which, instead of choosing folds at random from the data, we choose monophyletic clades on the phylogeny. By estimating our error on groups that can be considered to have evolved independently of the data on which the model was trained, we prevented confounding the effects of phylogenetic structure from influencing our model accuracy estimates.

In practice this meant building a series of random forest models for a given phylogeny, each leaving out a single class (blocked cross validation with classes as the folds). We then estimated our error by predicting each of the excluded folds in turn and calculating metrics of model performance. We repeated this process for each phylum.

To assess how well we could predict generalism when extrapolating across phyla (as opposed to within phyla, as above), we took a similar approach, this time considering all phyla together in a blocked cross validation framework, and using each phylum as a fold. We then assessed cross-phylum performance by predicting the generalism of species in one phylum using a model trained on the three other phyla, repeating for each phylum in turn.

#### Phylogenetic logistic regression to test for significant associations

We followed up our random forest analysis of generalism with a formal correction for phylogeny. We obtained the most important traits that predicted generalism by building a random forest on each phylum individually, and then selecting the top five ranked traits for each model based on mean decrease in the accuracy of the model when that variable’s values are permuted. We then performed phylogenetic logistic regression to predict generalism based on each of these traits for each of the relevant phyla (20 tests, phylolm R package; [51]). Following the recommendation of Ives and Garland based on our small sample size [52], we report bootstrapped *p* values (10,000 bootstraps).

### Metabolic breadth versus trait associations

We predicted metabolic breadth of taxa using a similar approach to that used for prediction of generalism. Specifically, we defined metabolic breadth of a taxon as the number of carbon substrates on which a microbe can grow as recorded in our database. We then attempted to predict this number using all other traits (excluding substrate use traits). We used a blocked-cross validation approach with phyla as folds, and built random forests for regression (randomForest R package, 5000 trees).

### Model assessment

We used Cohen’s $\kappa$ [53] as our index of model performance. Briefly, this index measures the increase in predictive performance over a null model that has information only about class prevalence. If classes are highly unbalanced, it is easy to acheive high accuracy with little discriminative ability by always guessing the prevalent class. Cohen’s $\kappa$ essentially corrects for this problem. Values greater than zero indicate discriminative ability greater than this null model, whereas negative values indicate the opposite (e.g., randomly guessing “yes” 50% of the time when the actual prevalence is “yes” 90% of the time). As noted above, in order to get an unbiased/phylogenetically corrected estimate of model performance we used blocked-cross validation, predicting the quantity of interest (source site, generalism, metabolic breadth, etc.) for each phylum in turn on the basis of the others and taking the mean $\kappa$ across these models.

## Supplementary Information

**Additional file 1.** Trait Database.

**Additional file 2.** Supplemental Table and Figures.

## Data Availability

All data generated or analysed during this study are included in this published article.

## Abbreviations

pH: Potential of hydrogen
FDR: False discovery rate
RMSE: Root mean squared error
NCBI: National Center for Biotechnology Information
HMP: Human Microbiome Project
EBIC: Extended Bayesian information criterion
