# Supplementary material for: Exploring the functional composition of the human microbiome using a hand-curated microbial trait database
Source: BMC Bioinformatics. 2021 Jun 7;22:306. doi: 10.1186/s12859-021-04216-2 (PMC8186035; doi:10.1186/s12859-021-04216-2)
Supplement: Supplementary file 2 — Additional file 2. Supplemental Table and Figures. [file 12859_2021_4216_MOESM2_ESM.pdf]

Supplement: Exploring the Functional Composition of the Human Microbiome Using a Hand-Curated Microbial Trait Database

March 14, 2021

	Test					
	Actinobacteria	Bacteroidetes	Firmicutes	Proteobacteria	Mean	# Traits
Stool	0.489	0.042	0.922	0.017	0.368	31
Posterior Fornix	0.488	0.314	-0.004	0.264	0.265	26
Anterior Nares	0.008	-0.016	-0.020	0	0	19
Retroauricular Crease	0.148	-0.035	0.128	0.029	0.067	21
Tongue Dorsum	0	0	0.106	-0.024	0.021	21
Supragingival Plaque	0	0	0.314	0	0.079	23
Buccal Mucosa	0	0	-0.029	-0.008	-0.009	26
Mouth (All)	0.011	0	0.708	0.773	0.373	24

S1 Table: Cohen's κ for predicting sample source site, using only traits with a quartile coefficient of dispersion less than 0.5 among phyla in that site. This table presents results for analyses restricted to traits that vary relatively little among phyla, at the cost of throwing out the information held in the ommited traits (number of traits included in analysis for each site shown in last column).

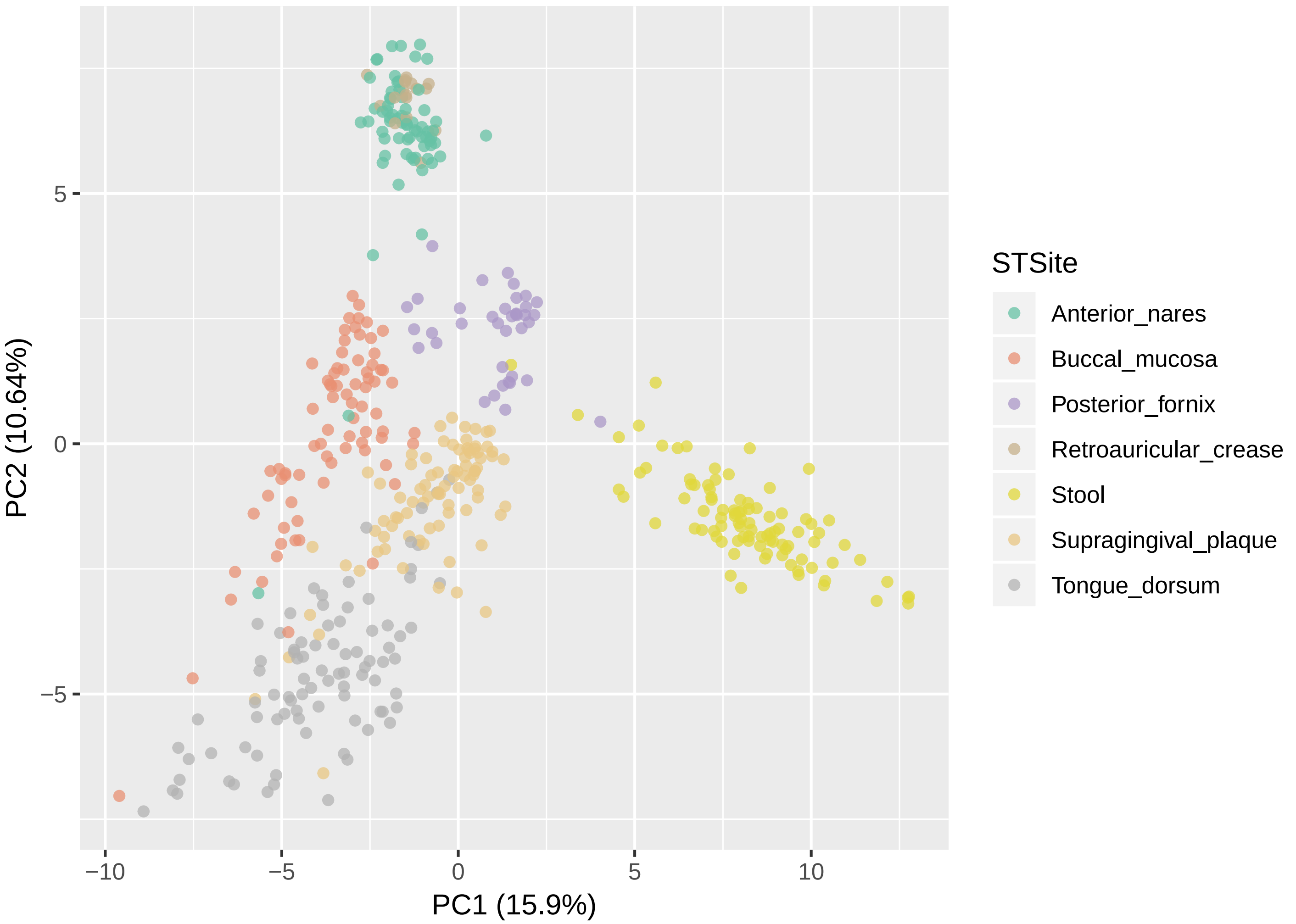

S1 Fig: PCA for each sample based on its centered and scaled mean trait values (weighted by taxon abundance). The top-loaded variables on the first principle component are use of arabinose (0.18), use of xylose (0.17), genome length (0.17), α-galactosidase activity (0.17), and use of rhamnose (0.17). The top-loaded variables on the second principle component are minimum NaCl (0.21), use of glucose (0.20), maximum NaCl (0.18), use of mannose (0.16), and use of galactose (0.16).

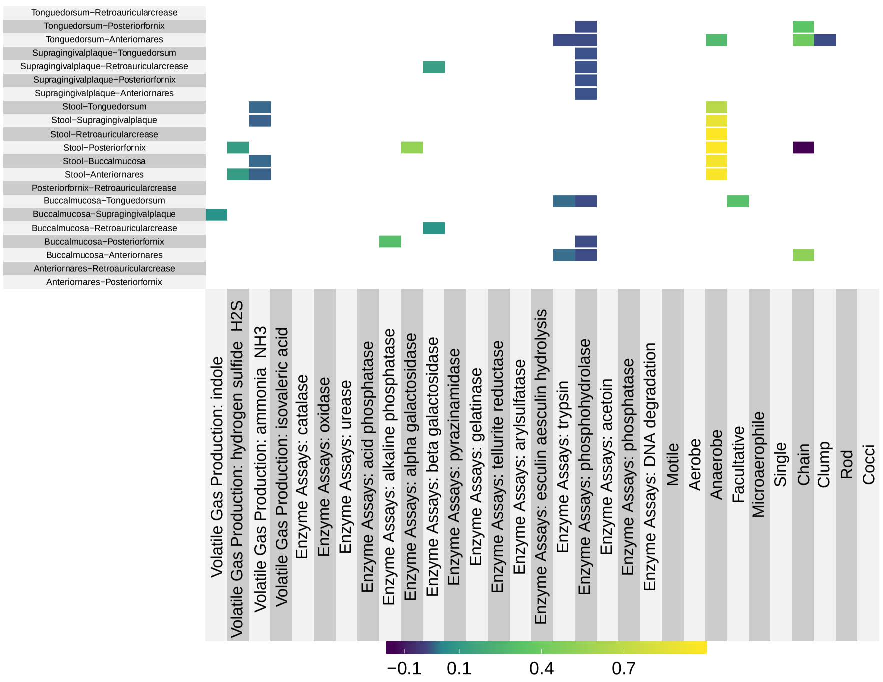

(a)

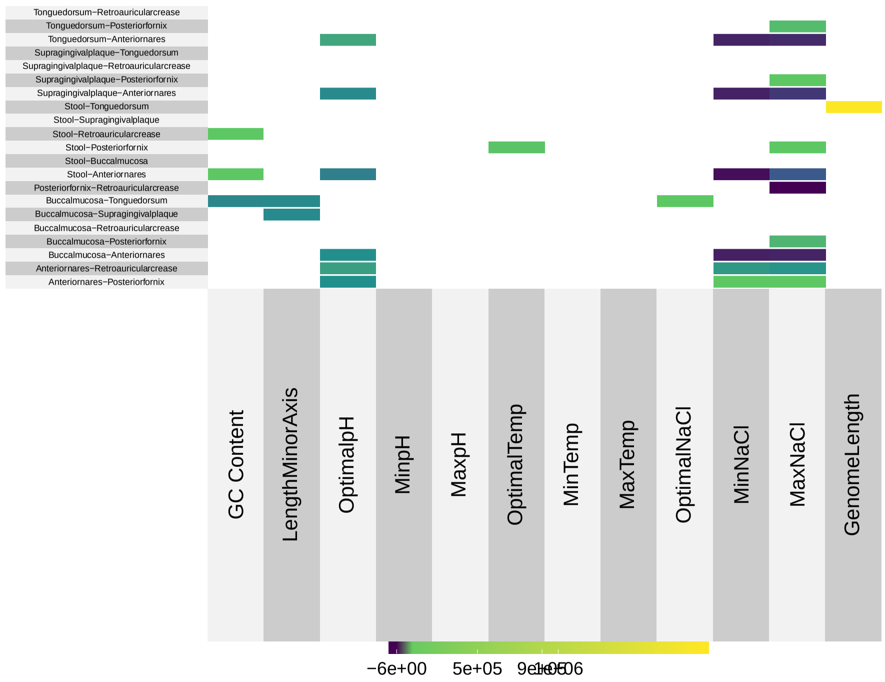

(b)

S2 Fig: Pairwise differences in trait values between body sites shown (difference in means weighted by taxon abundance). Interactions that were not individually significant in all phyla are left blank. Traits separated into categories for readability: (a) qualitative with categorical values (split into dummy variables for multi-level traits) and (b) quantitative with continous values. For carbon substrate use traits see Fig S3 Fig.

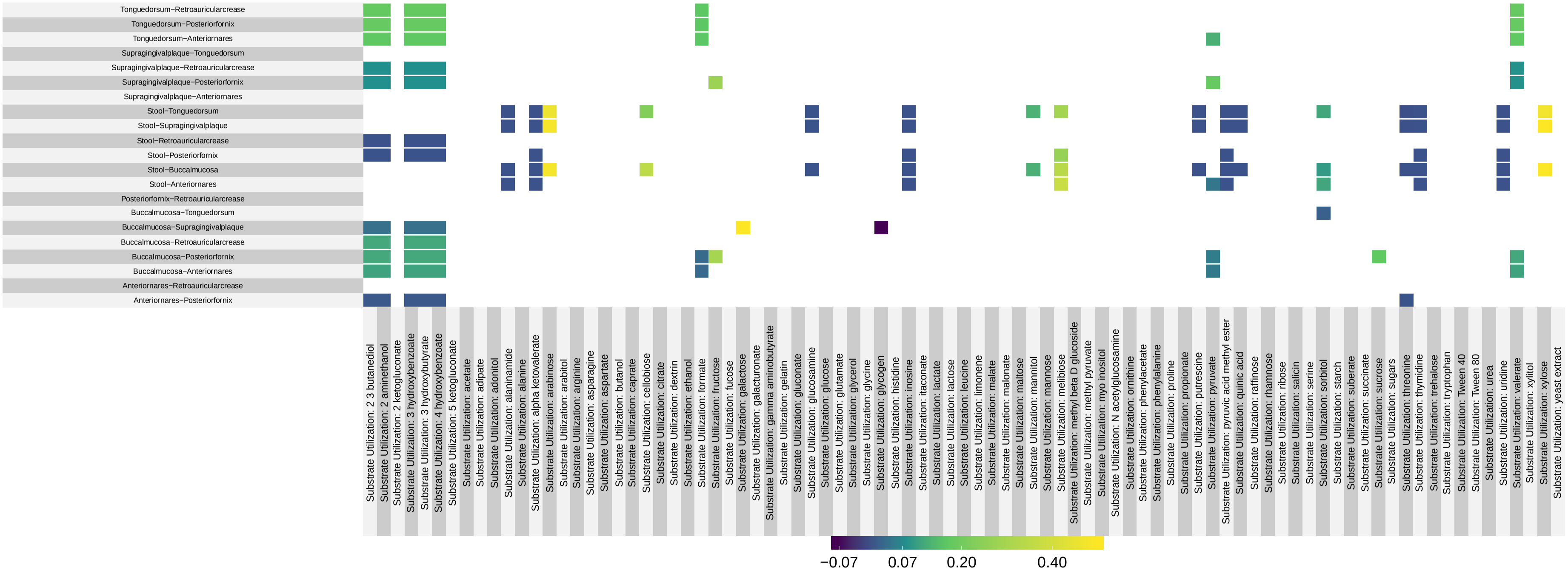

S3 Fig: Pairwise differences in carbon substrate use frequency between body sites shown (difference in means weighted by taxon abundance). Interactions that were not individually significant in all phyla are left blank. Shown here are binary traits indicating the ability to grow on specific carbon sources. For other traits see Fig S2 Fig.

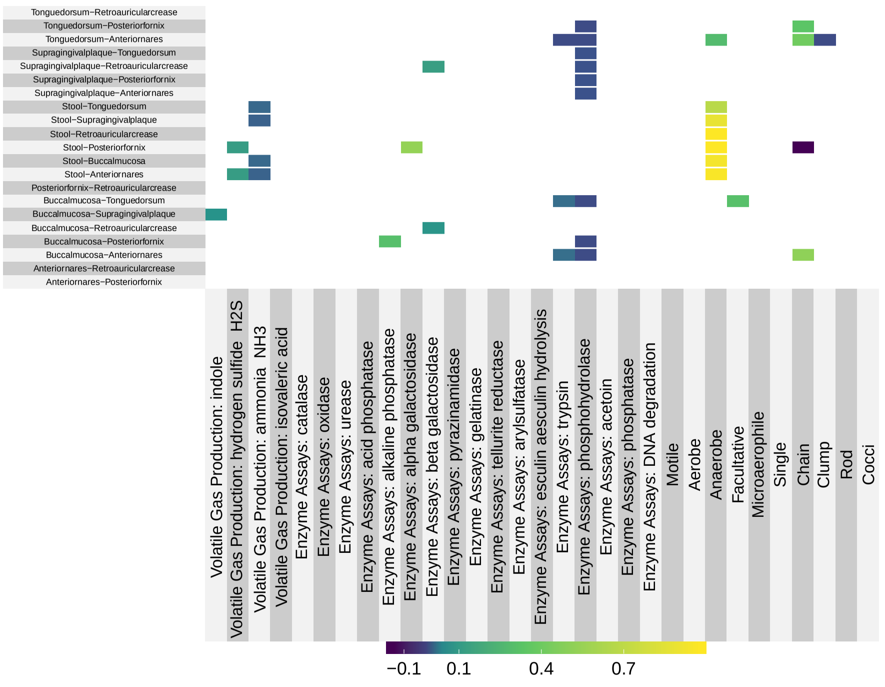

(a)

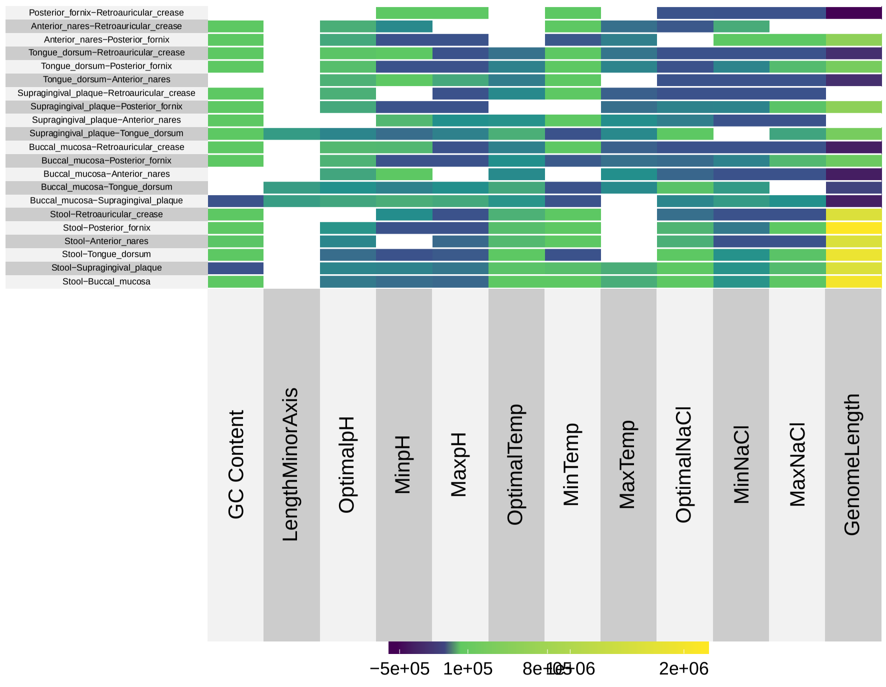

(b)

S4 Fig: Pairwise differences in trait values between body sites shown (difference in means weighted by taxon abundance). Interactions that were not significant are left blank. Traits separated into categories for readability: (a) qualitative with categorical values (split into dummy variables for multi-level traits) and (b) quantitative with continous values. For carbon substrate use traits see Fig S5 Fig.

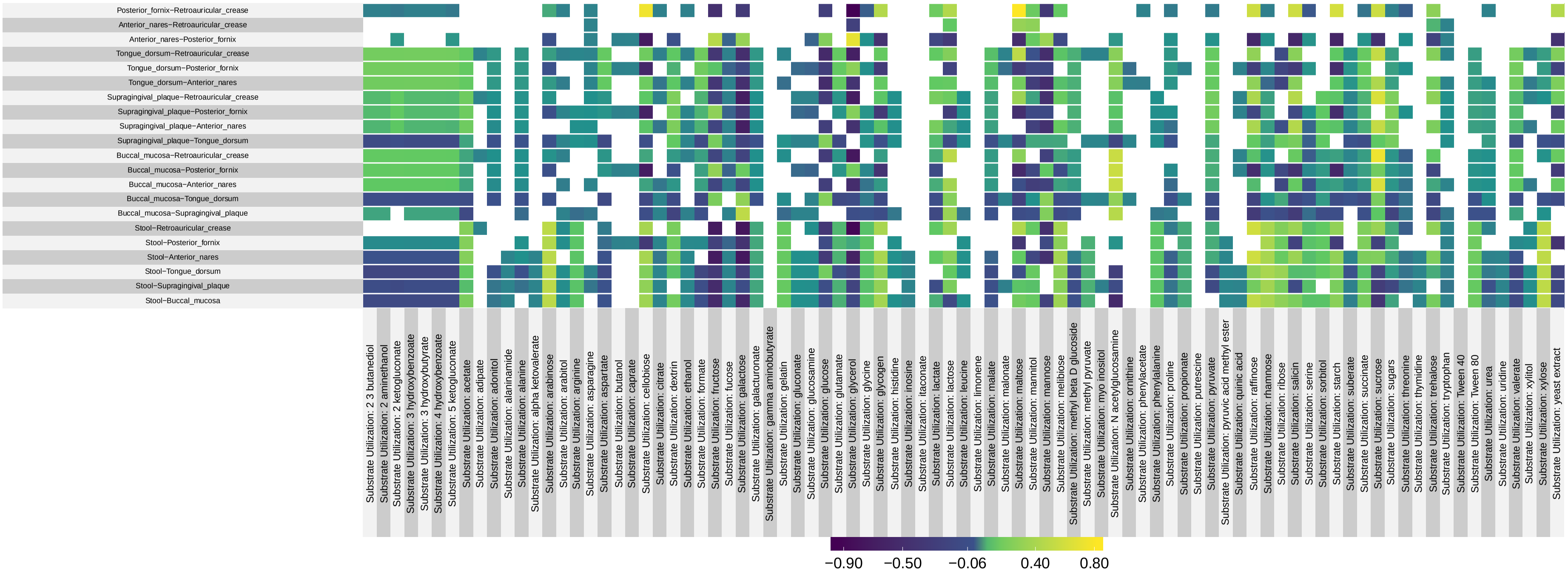

S5 Fig: Pairwise differences in carbon substrate use frequency between body sites shown (difference in means weighted by taxon abundance). Interactions that were not significant are left blank. Shown here are binary traits indicating the ability to grow on specific carbon sources. For other traits see Fig S4 Fig.

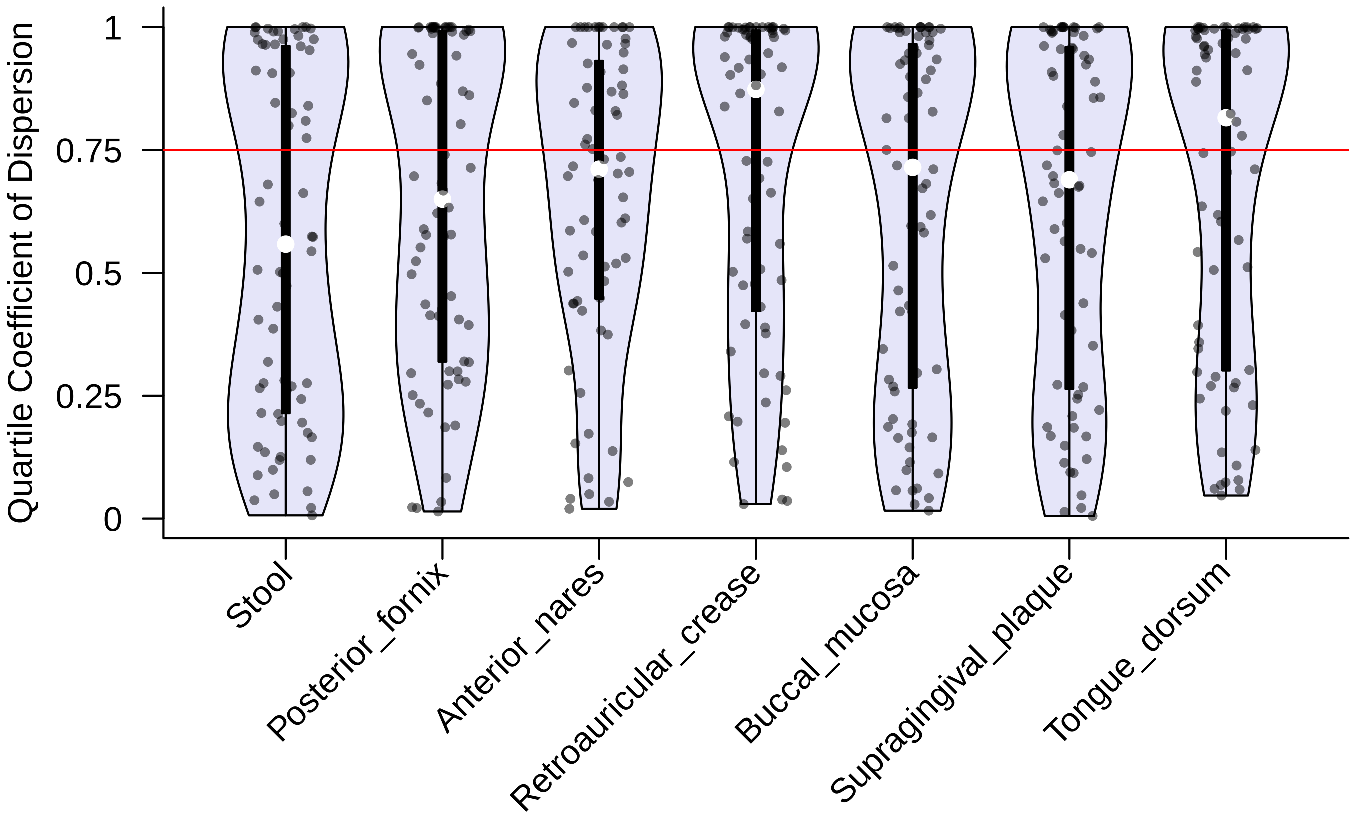

S6 Fig: Variability of trait scores across phyla in each site. Each point represents an individual trait's quartile coefficient of dispersion (a measure of variability) of mean trait prevalence across phyla in a given site.

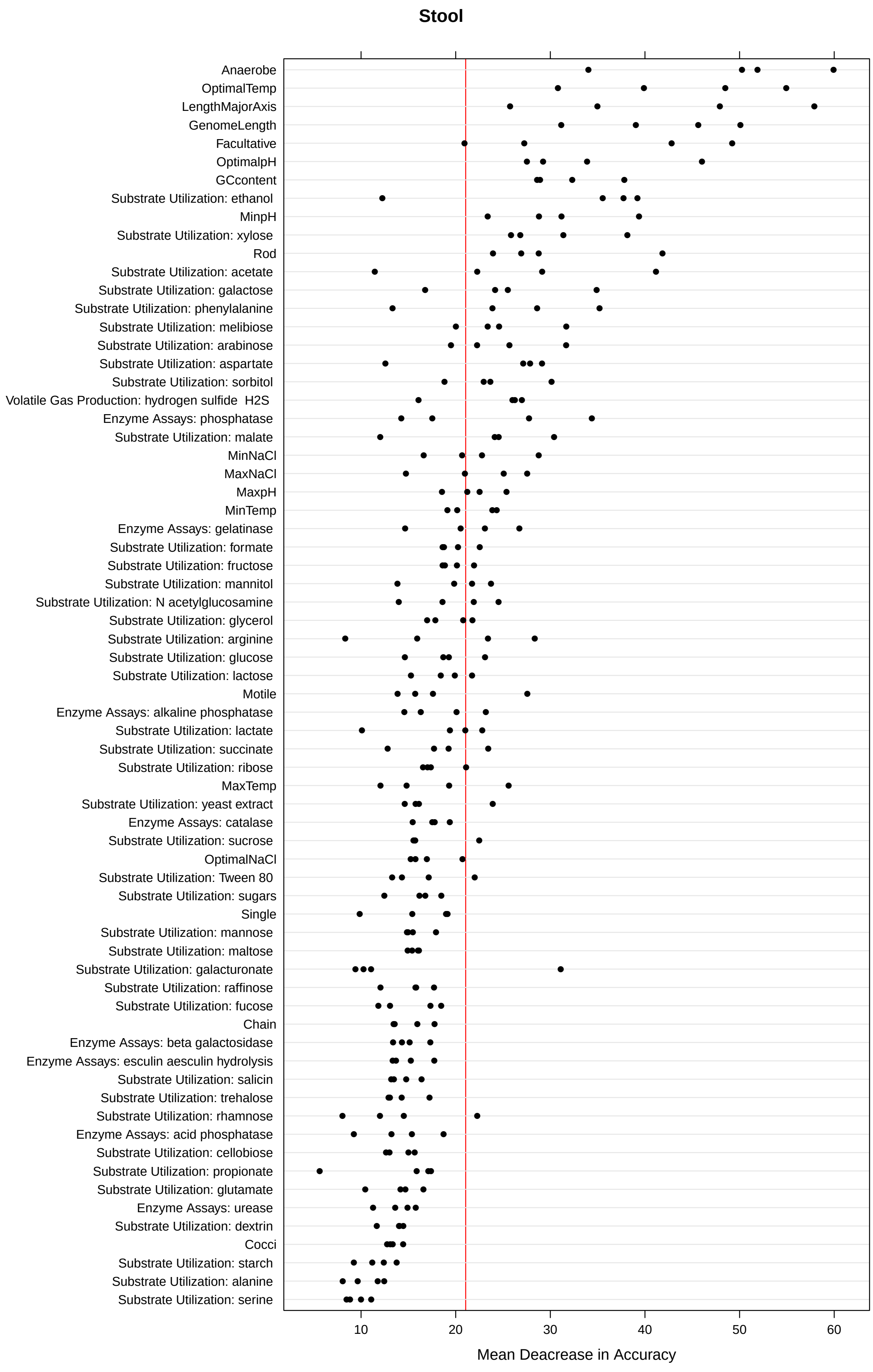

S7 Fig: Variable importance scores for the predictive models of whether a sample comes from the stool. Models performance is listed in Table 1. Each point indicates the score for a model built with a different validation fold (different test phyla).

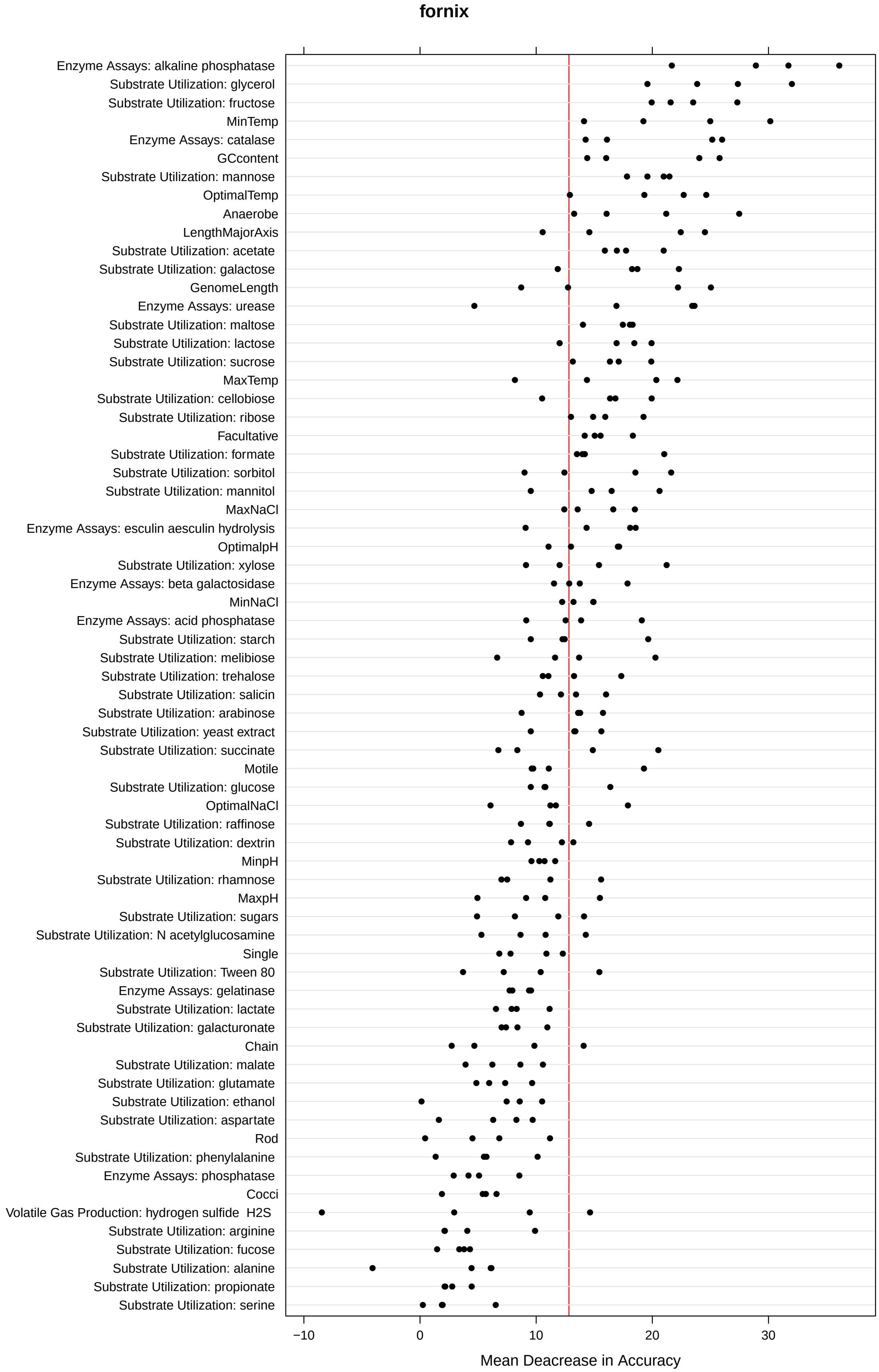

S8 Fig: Variable importance scores for the predictive models of whether a sample comes from the posterior fornix. Models performance is listed in Table 1. Each point indicates the score for a model built with a different validation fold (different test phyla).

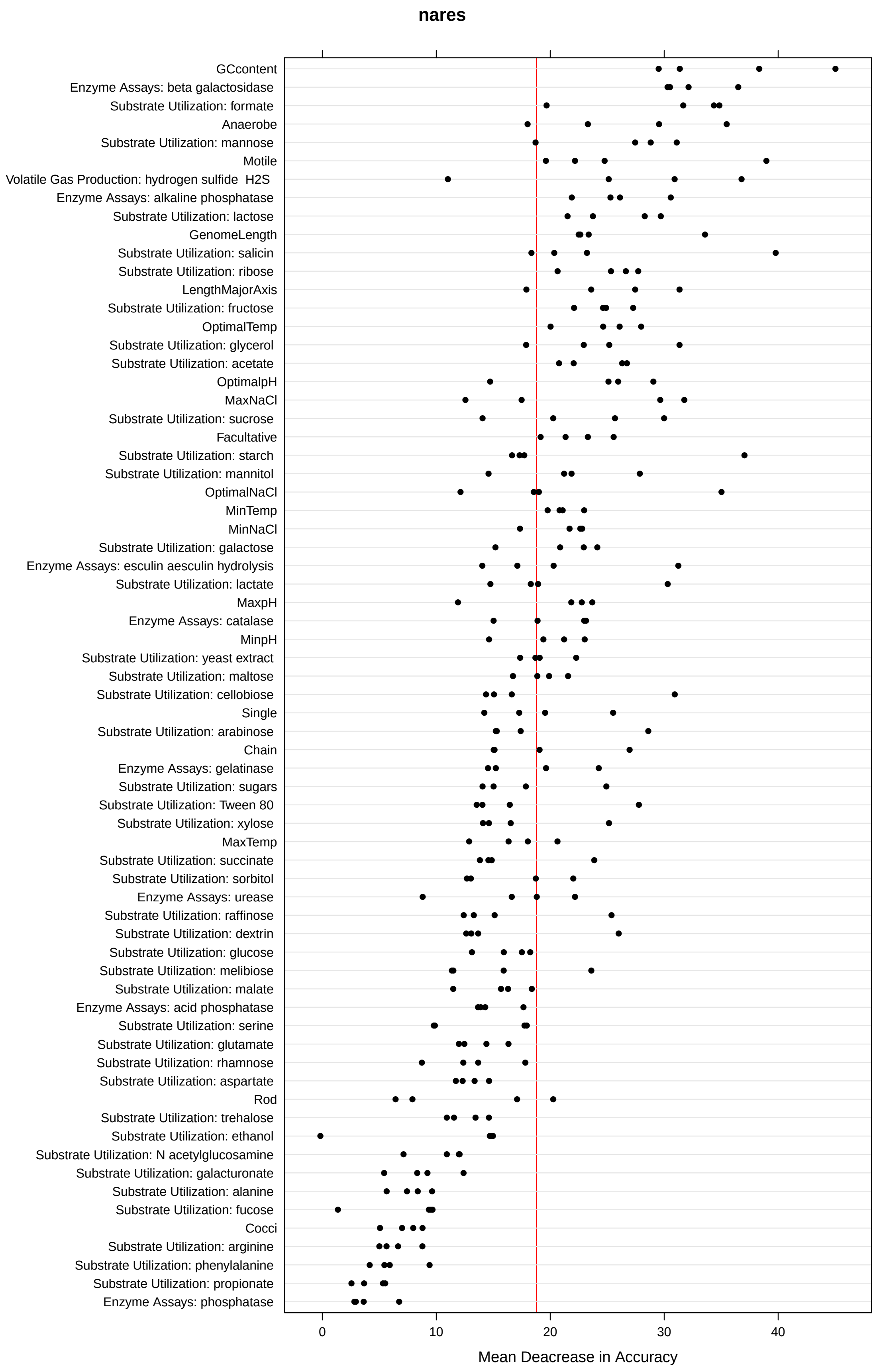

S9 Fig: Variable importance scores for the predictive models of whether a sample comes from the anterior nares. Models performance is listed in Table 1. Each point indicates the score for a model built with a different validation fold (different test phyla).

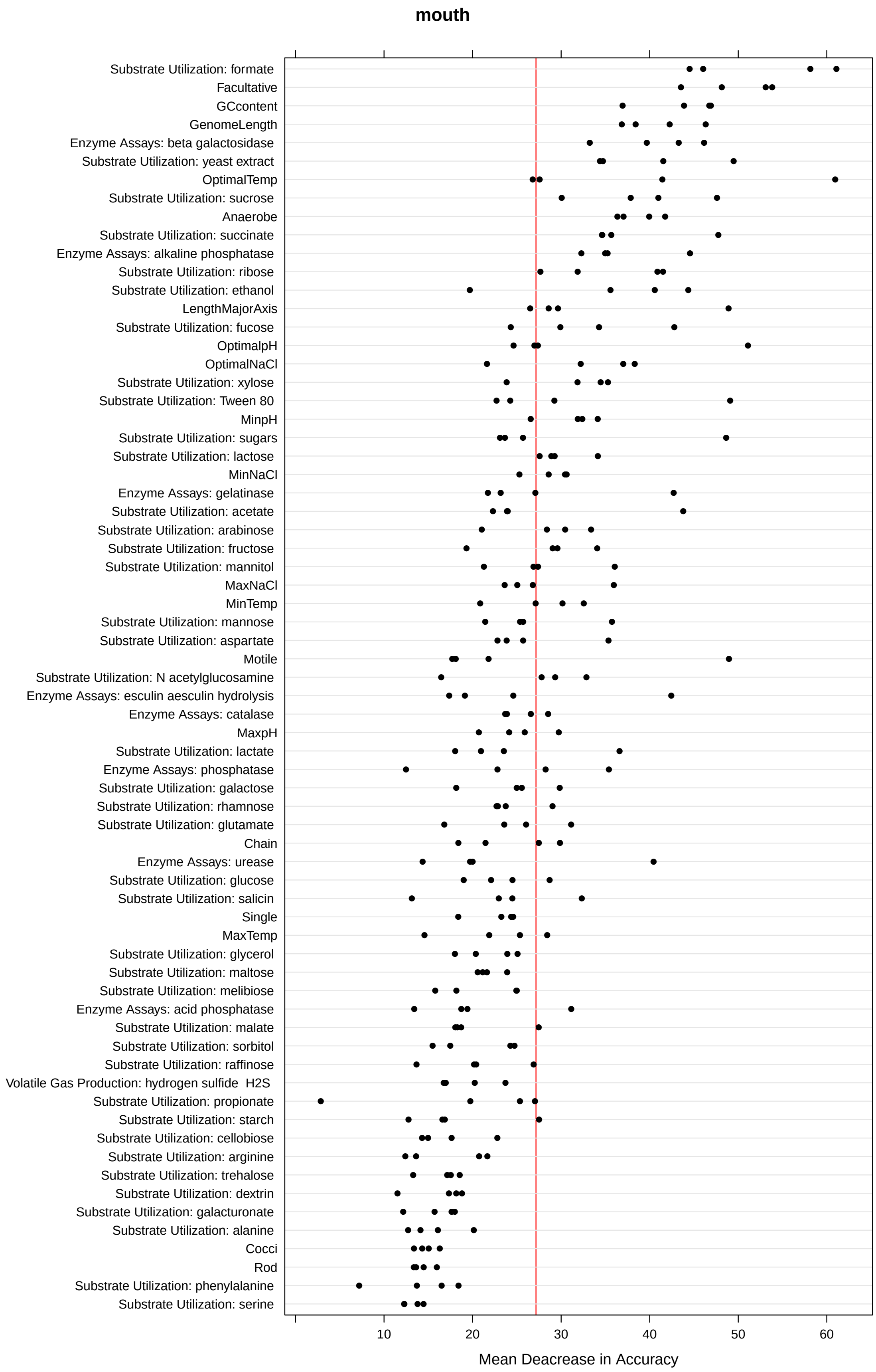

S10 Fig: Variable importance scores for the predictive models of whether a sample comes from the mouth. Models performance is listed in Table 1. Each point indicates the score for a model built with a different validation fold (different test phyla).

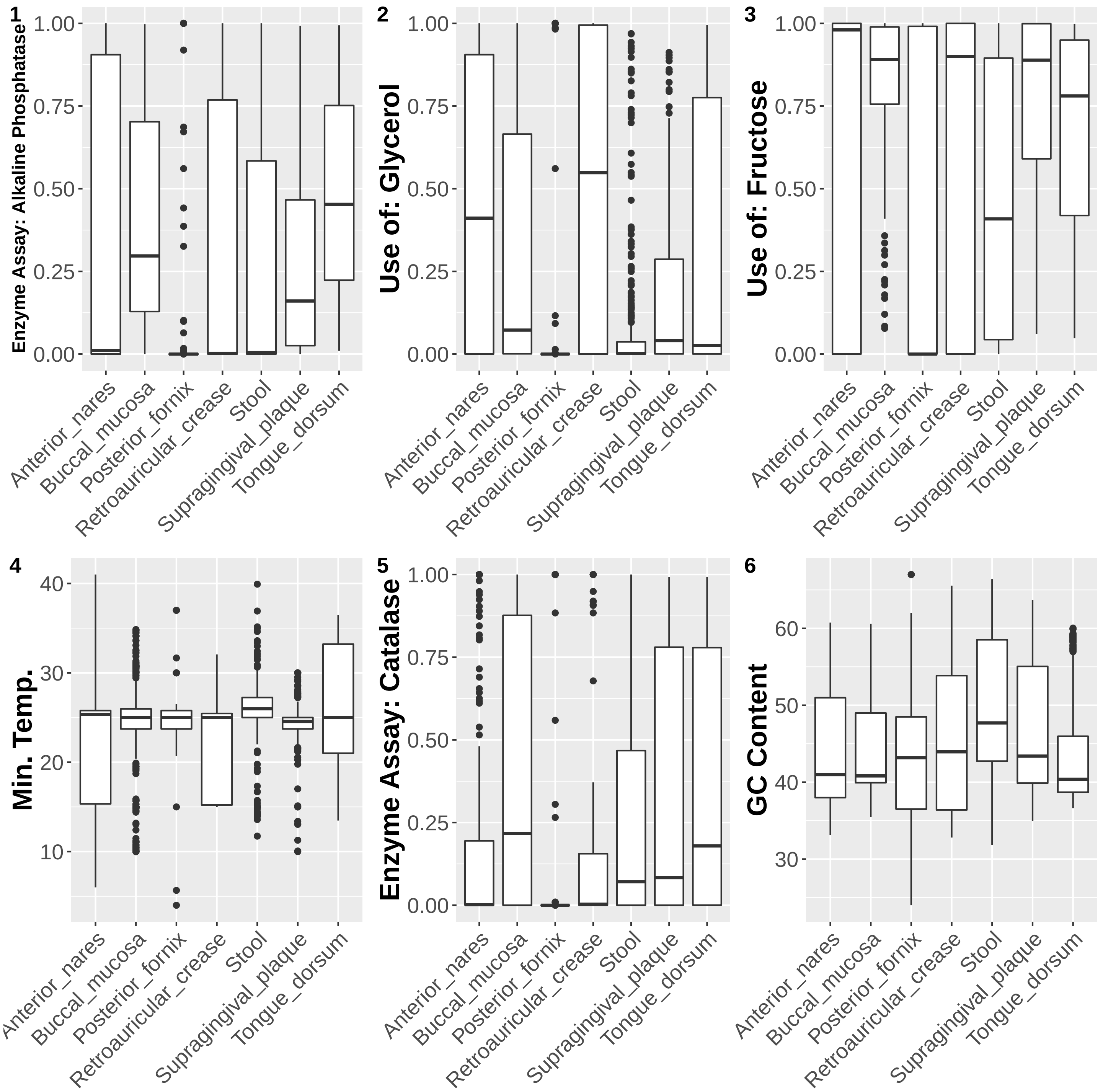

S11 Fig: Top predictors for posterior fornix with rank shown in upper left corner. Top predictors across phyla of sample source site, for which importance scores are above the average variable importance across all predictors for all four training sets (S8 Fig). Shown are mean trait values across all samples in the dataset, split up by body site.

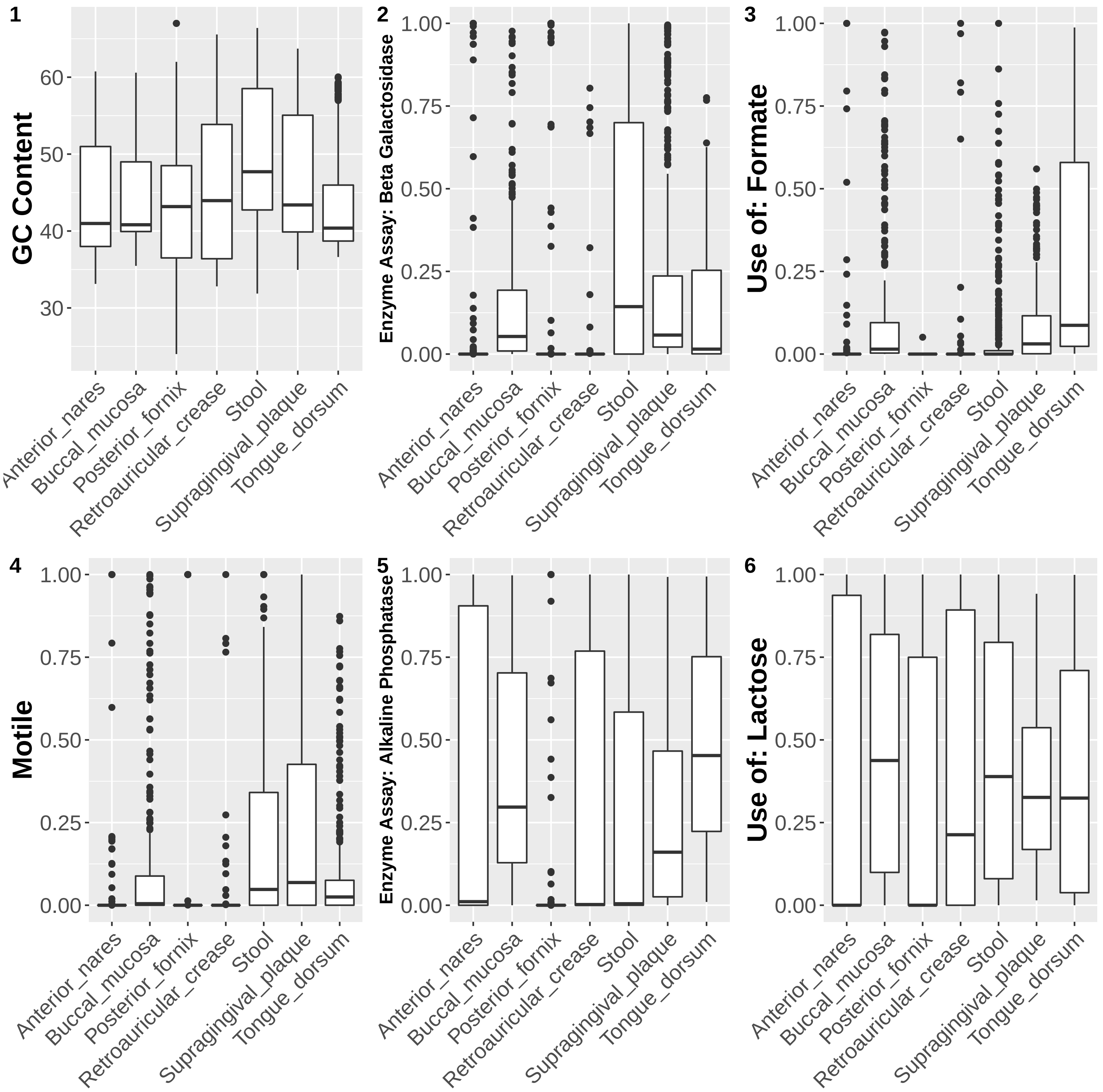

S12 Fig: Top predictors for anterior nares with rank shown in upper left corner. Top predictors across phyla of sample source site, for which importance scores are above the average variable importance across all predictors for all four training sets (S9 Fig). Shown are mean trait values across all samples in the dataset, split up by body site.

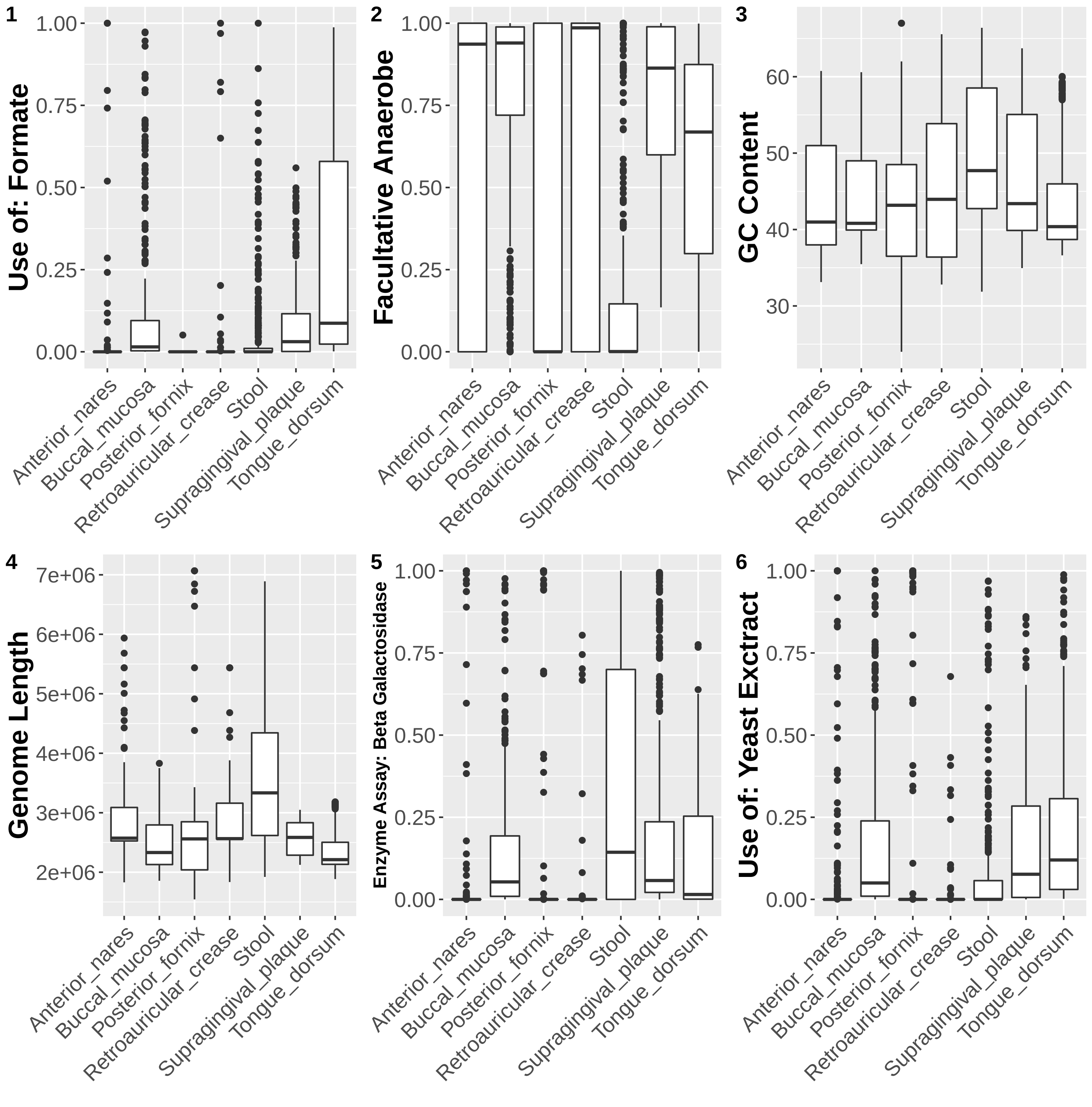

S13 Fig: Top predictors for mouth with rank shown in upper left corner. Top predictors across phyla of sample source site, for which importance scores are above the average variable importance across all predictors for all four training sets (S10 Fig). Shown are mean trait values across all samples in the dataset, split up by body site.

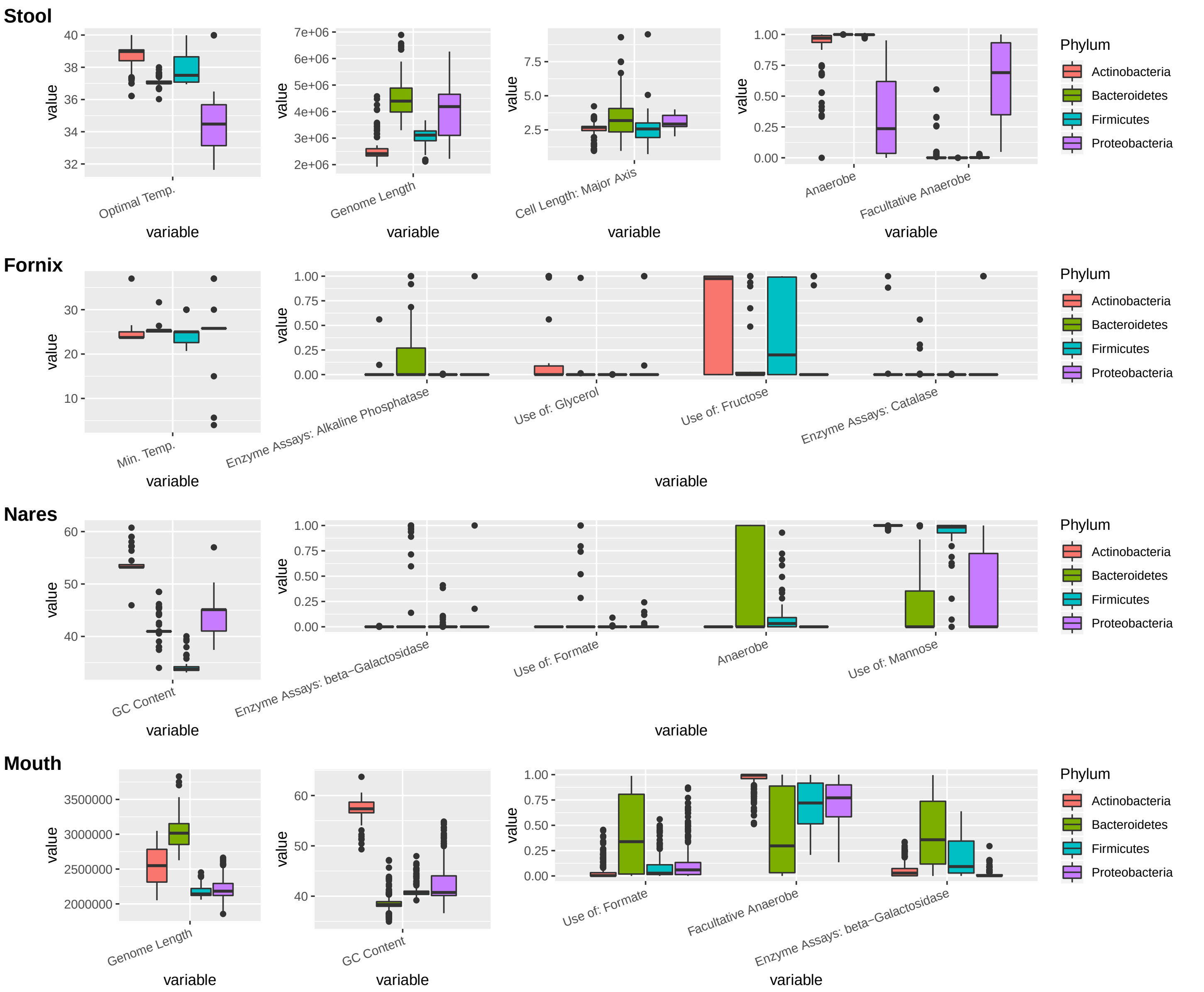

S14 Fig: Variability of top predictive traits across phyla for each site (top predictors for model with results in Table 1). Note that mouth appears to be more variable, and that different phyla appear to be the outlier across different traits. Shown are mean trait values across all samples in the dataset.

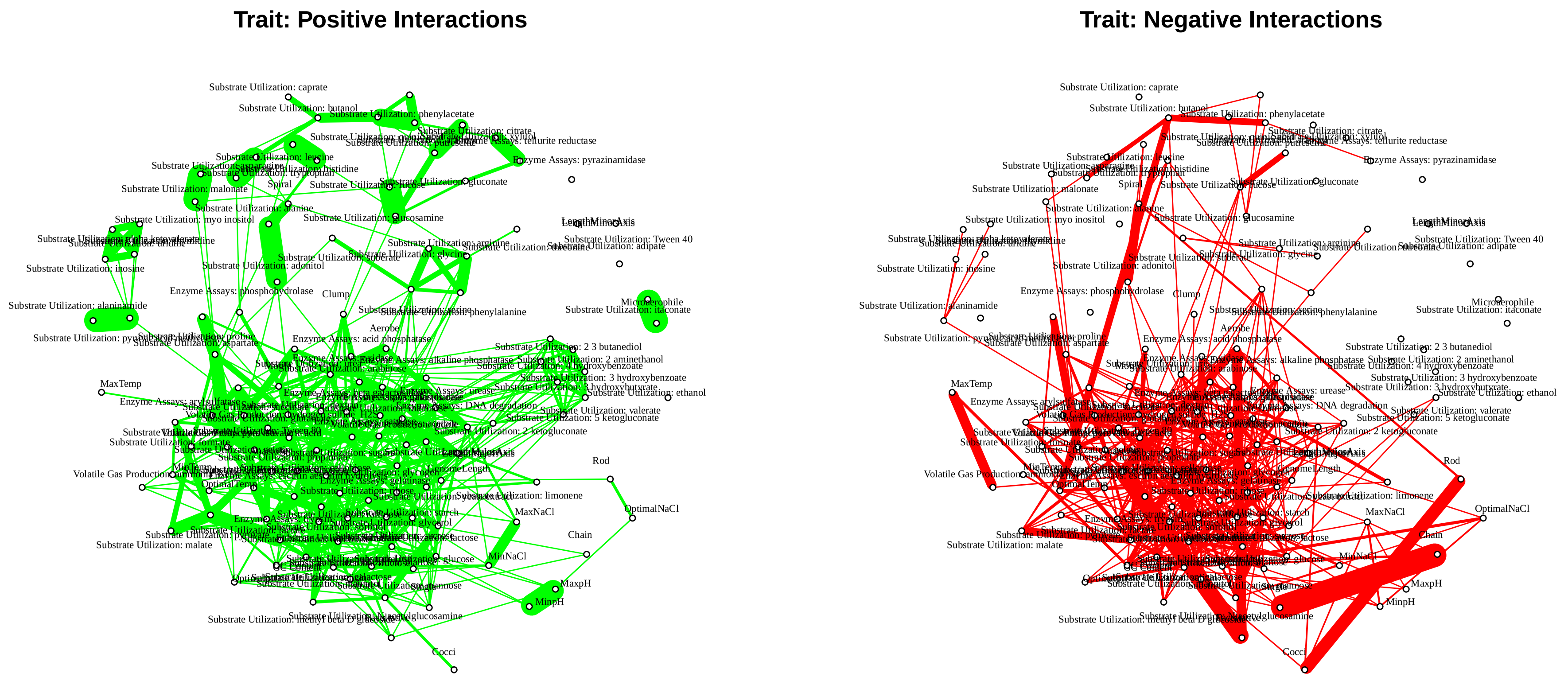

S15 Fig: Trait network inferred using the graphical lasso. Positive and negative edges (representing a positive or negative relationship between two traits) are shown separately for ease of viewing. The strength of an inferred interaction is indicated by the width of an edge.

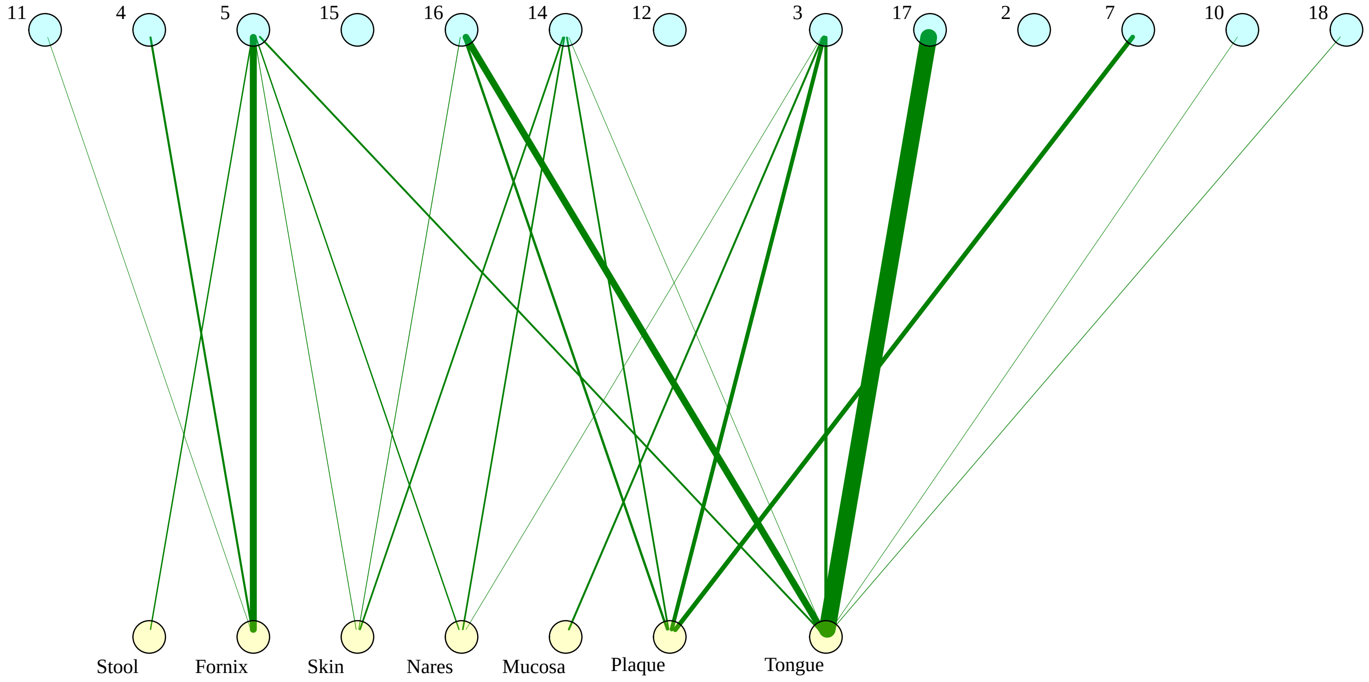

(a)

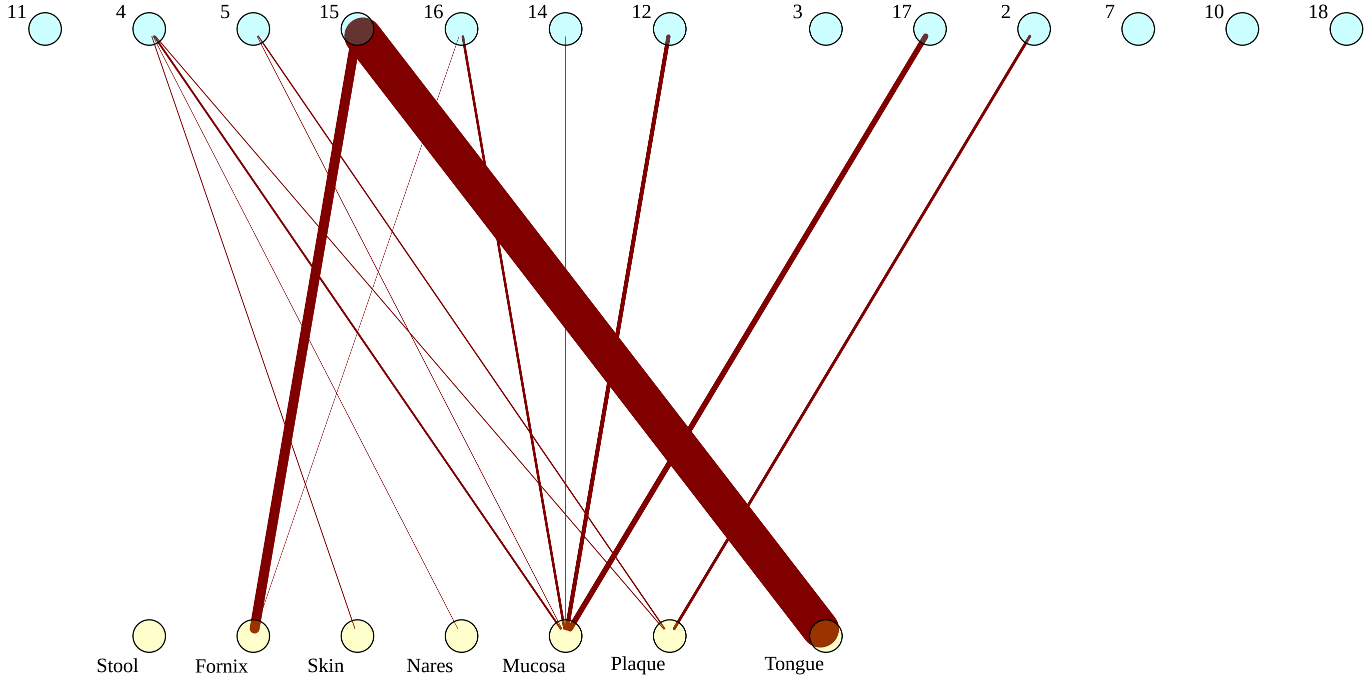

(b)

S16 Fig: Bipartite site-cluster network, where clusters are groups of traits that frequently co-occur. See Table 2 for a list of traits in each cluster. Identical information shown here as in Fig 4, but with (a) positive and (b) negative interactions separated out for ease of viewing.

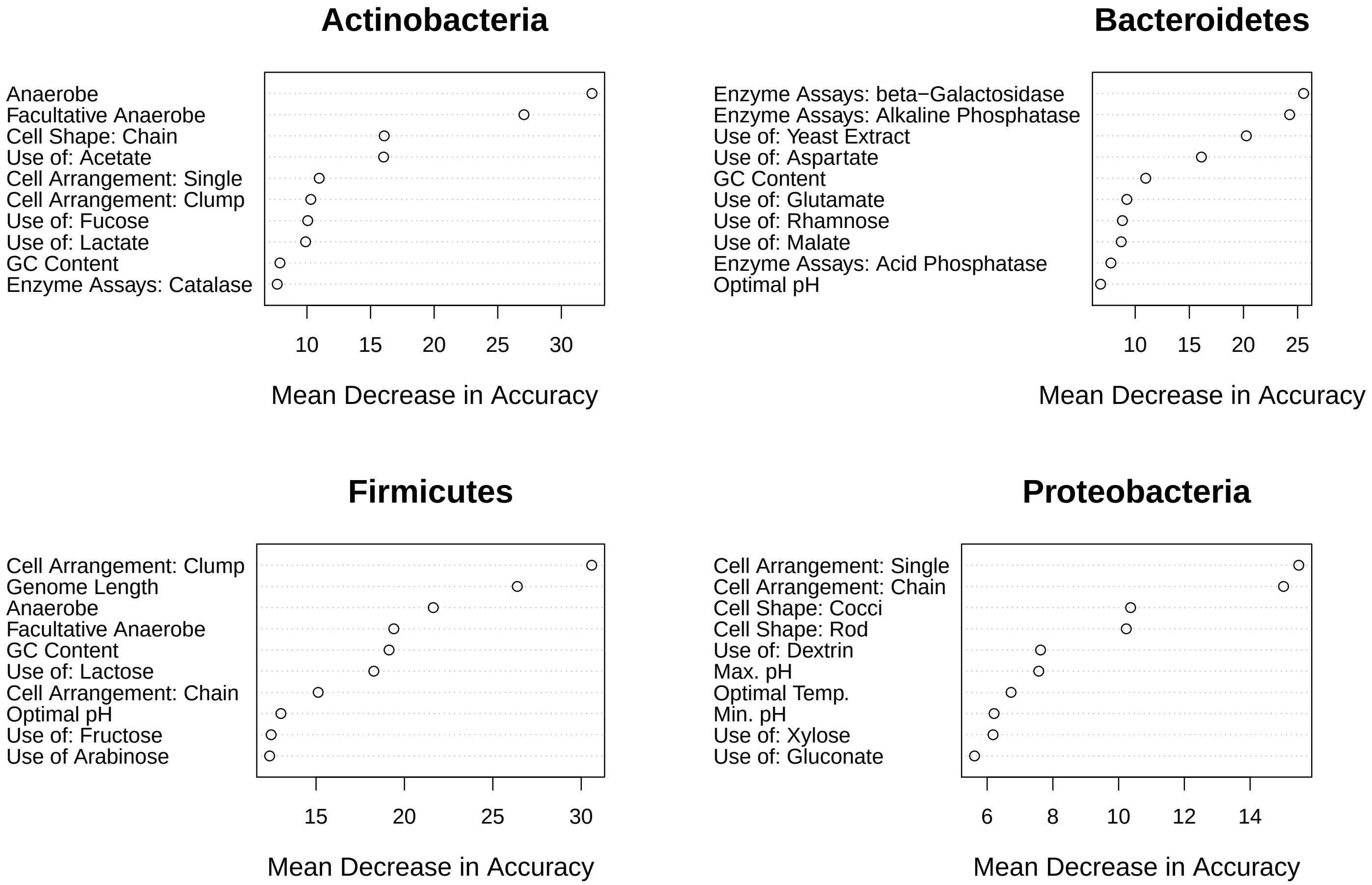

S17 Fig: Variable importance scores for random forest models of generalism built on individual phyla. Higher scores indicate that these traits were more important for predicting the generalism of species in a given phylum.

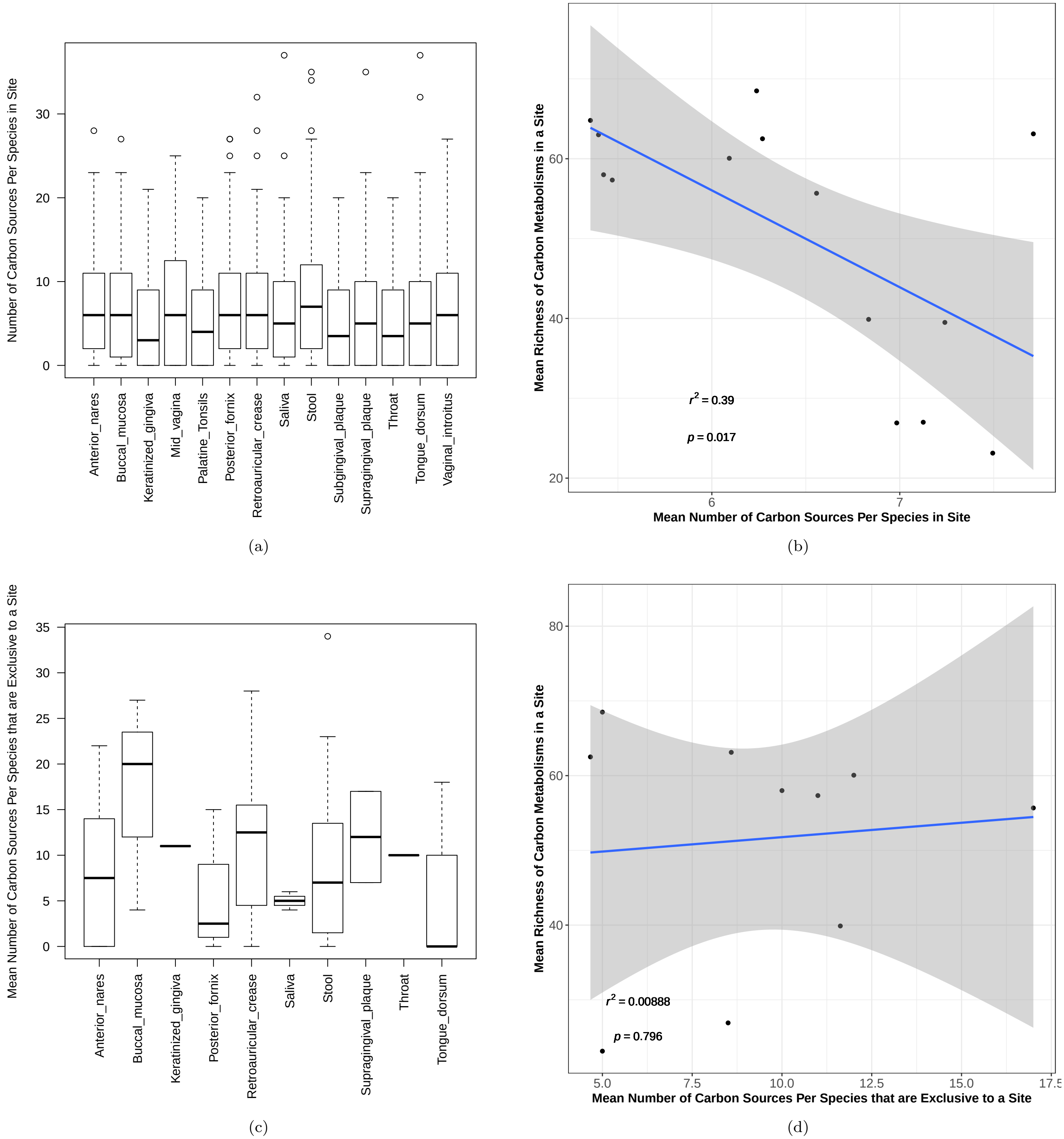

S18 Fig: Sites positioned along the alimentary tract (mouth and gut) do not harbor species that make use of a broader range of carbon substrates. (a,b) Number of carbon sources used by species found in any sample from a specific site. (c,d) Number of carbon sources used by species, only including species which are found exclusively in samples from a specific site.

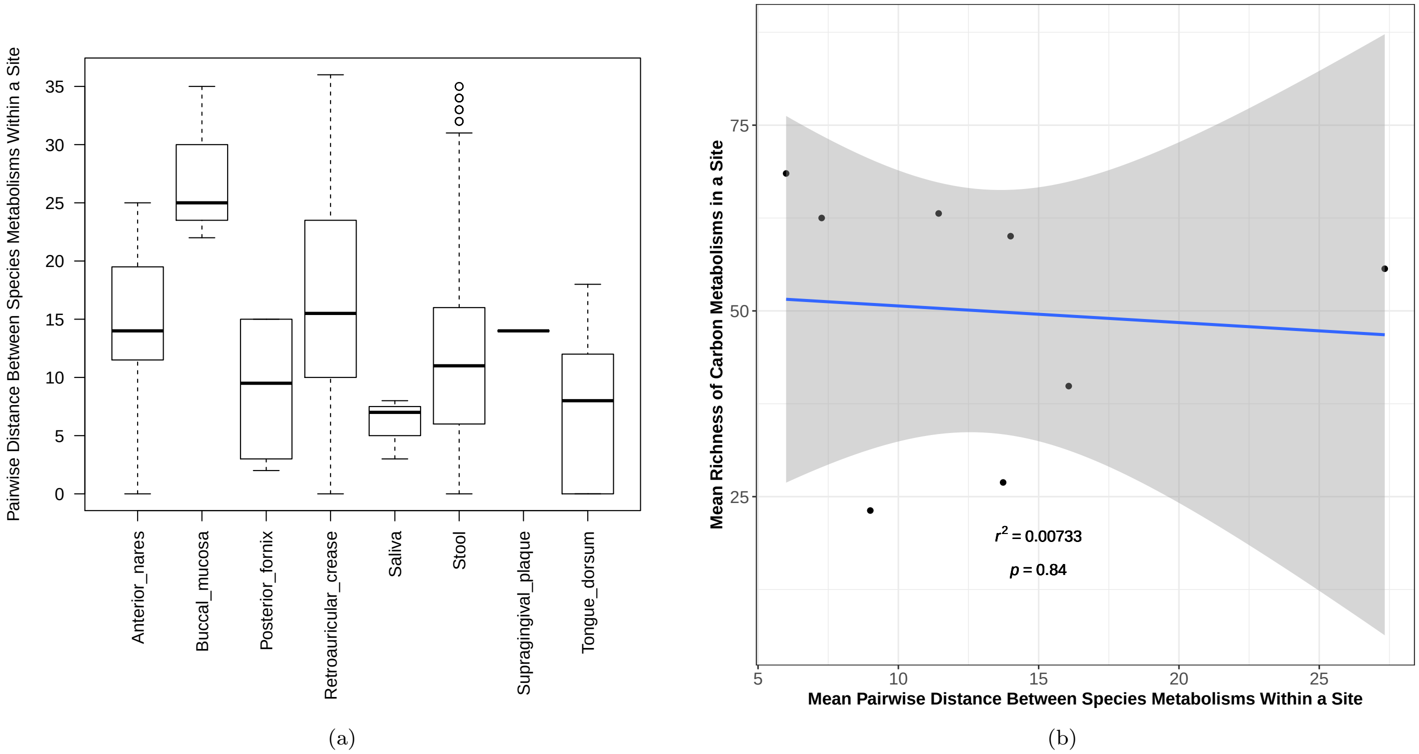

S19 Fig: How carbon substrates differ among species in sites. We tallied the number of carbon substrates used by each microbe to create (a) boxplots of pairwise distances (i.e., differences in the number of substrates used) and find (b) no relationship between the total number of carbon metabolisms represented at a body site and this measure of pairwise distance between species.

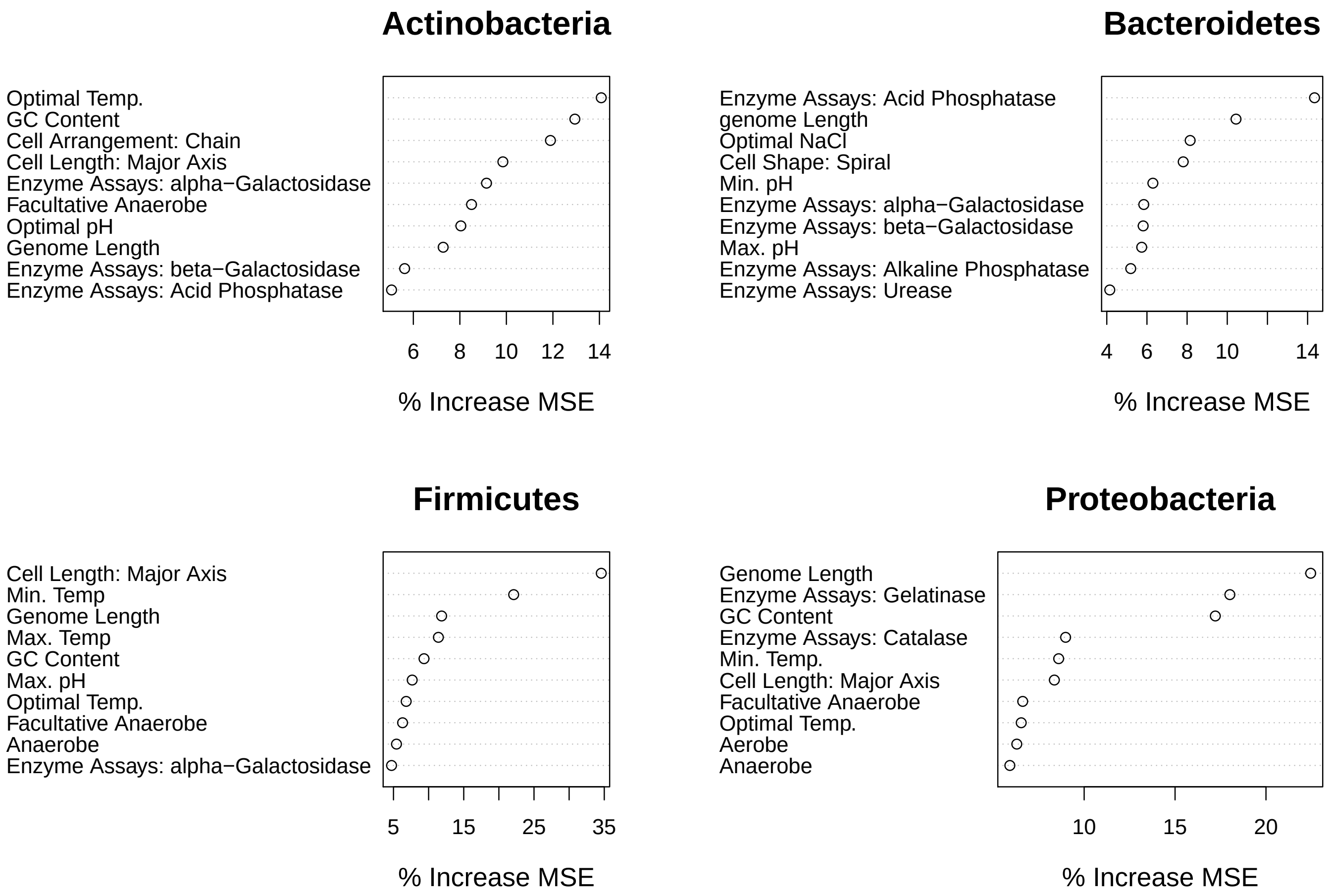

S20 Fig: Variable importance scores for random forests for regression built individually on each phylum to predict the number of carbon substrates a species can use. Higher scores indicate that these traits were more important for predicting the number of substrates of species in a given phylum.

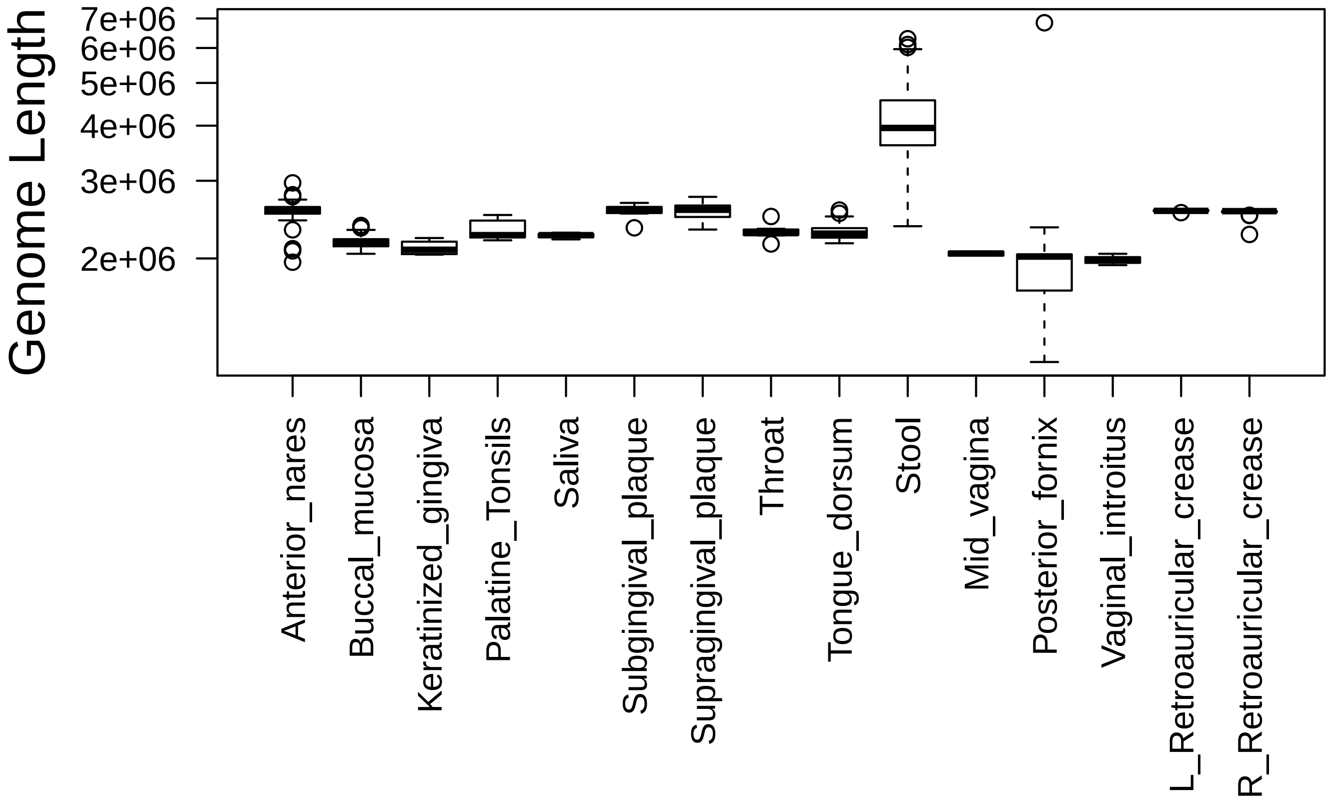

S21 Fig: Distributions of mean genome size of species in a sample sample across sites

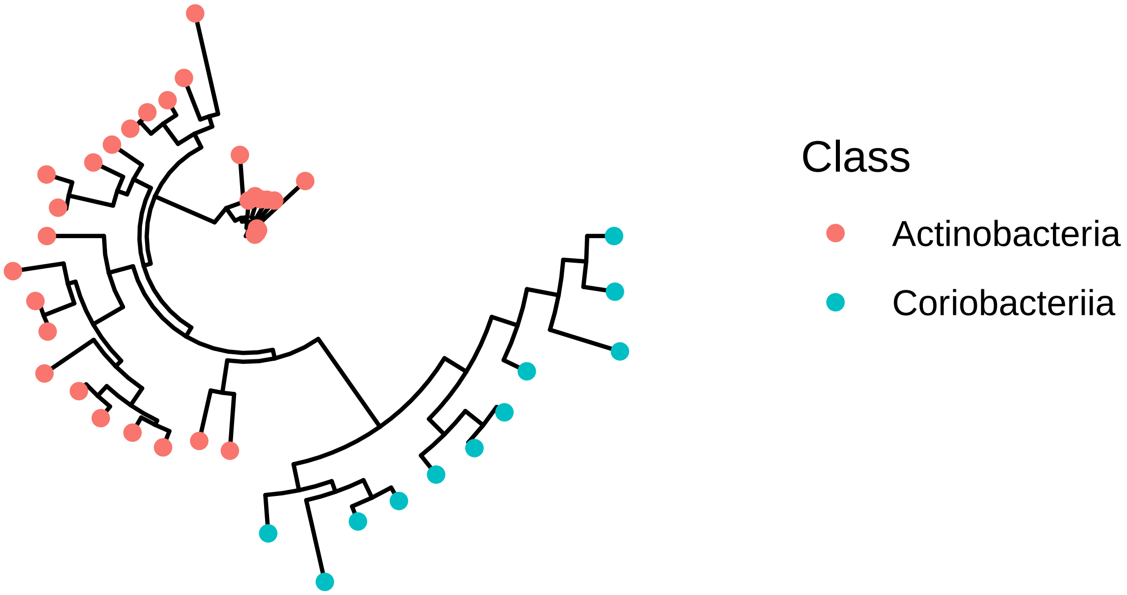

S22 Fig: Phylogeny of Actiobacteria.

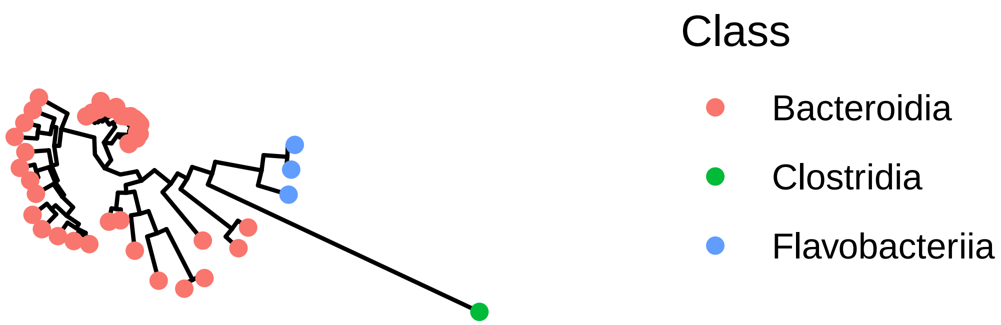

S23 Fig: Phylogeny of Bacteroidetes. Observe that Bacteroides pectinophilus is likely to be a member of the Firmicutes and NCBI Taxonomy lists its class as Clostridia, though it has yet to be reassigned officially to a new genus.

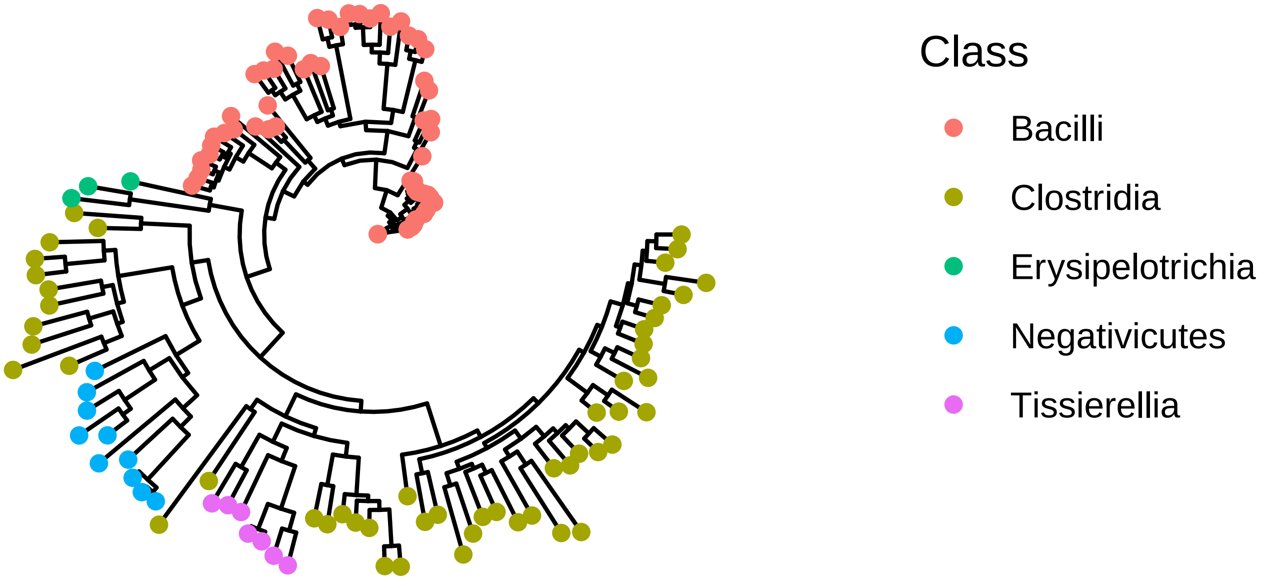

S24 Fig: Phylogeny of the Firmicutes. Note that the class Clostridia is non-monophyletic (as expected).

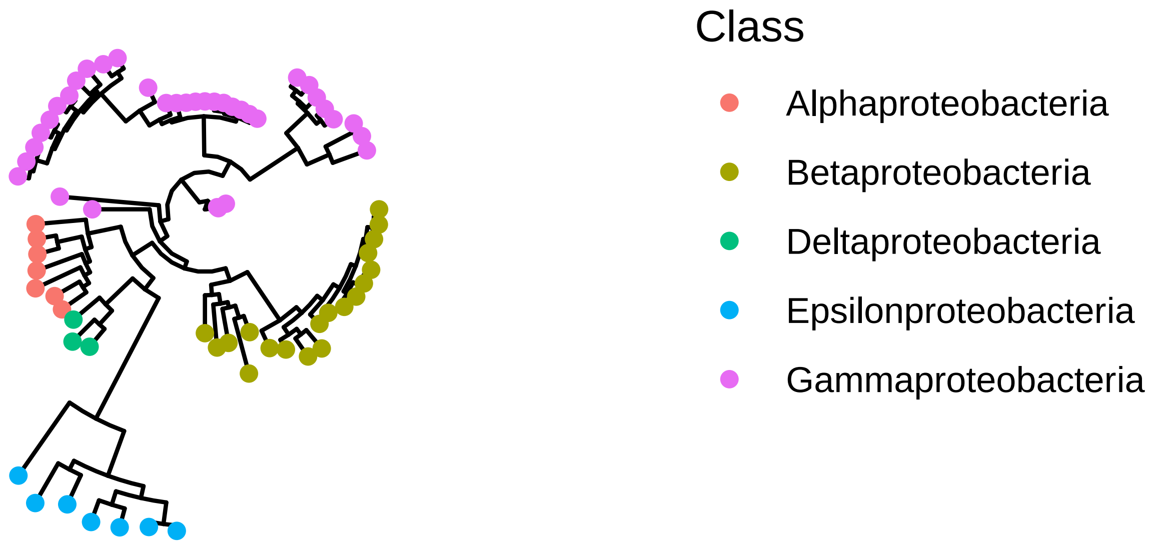

S25 Fig: Phylogeny of the Proteobacteria.

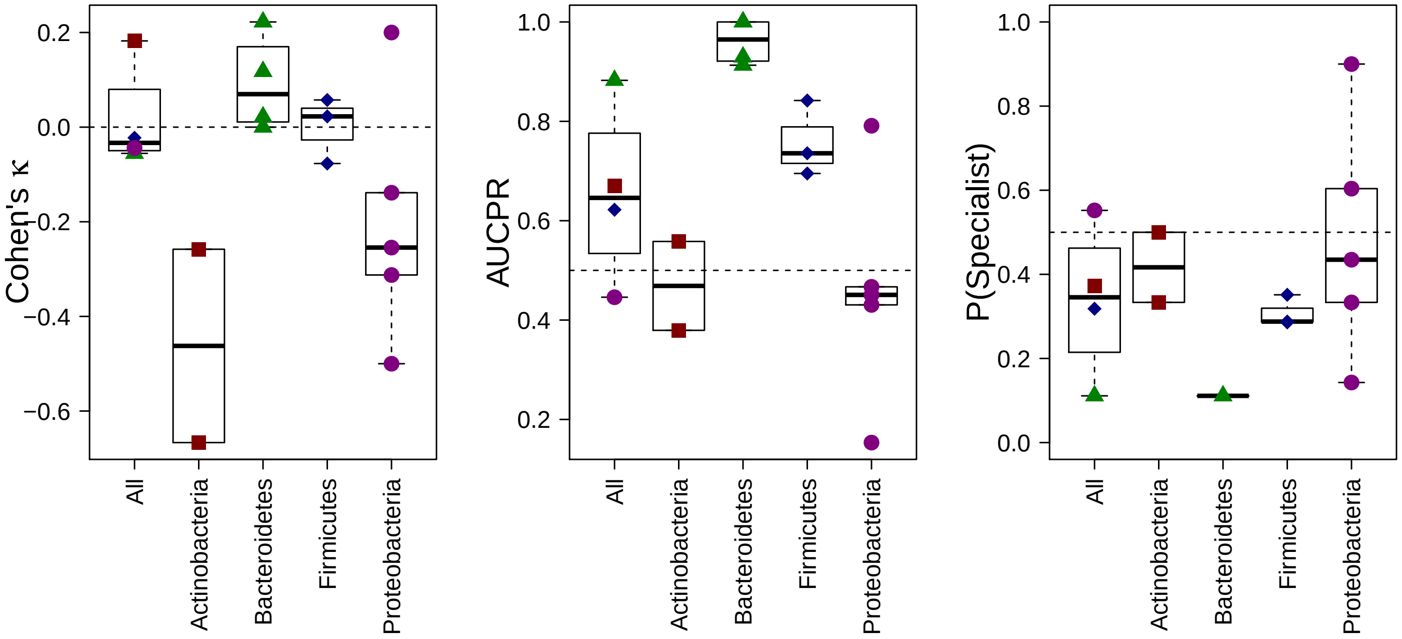

S26 Fig: Performance of random forest models predicting generalism inlcuding singletons (binary classification, present at more than one area or not). "All" means a blocked cross-validation with each phylum as a fold (Actinobacteria: red squares, Bacteroidetes: green triangles, Firmicutes: blue diamonds, Proteobacteria: purple circles). Within each phylum we performed blocked cross-validation using classes as folds, except in the case of Bacteroidetes where all species in the dataset were in the same class and order, so that families were used as the folds. Shown are two measures of performance (κ and area under the precision-recall curve), as well as the prevalence of specialist species in a fold (P(Specialist) for "probability is a specialist").

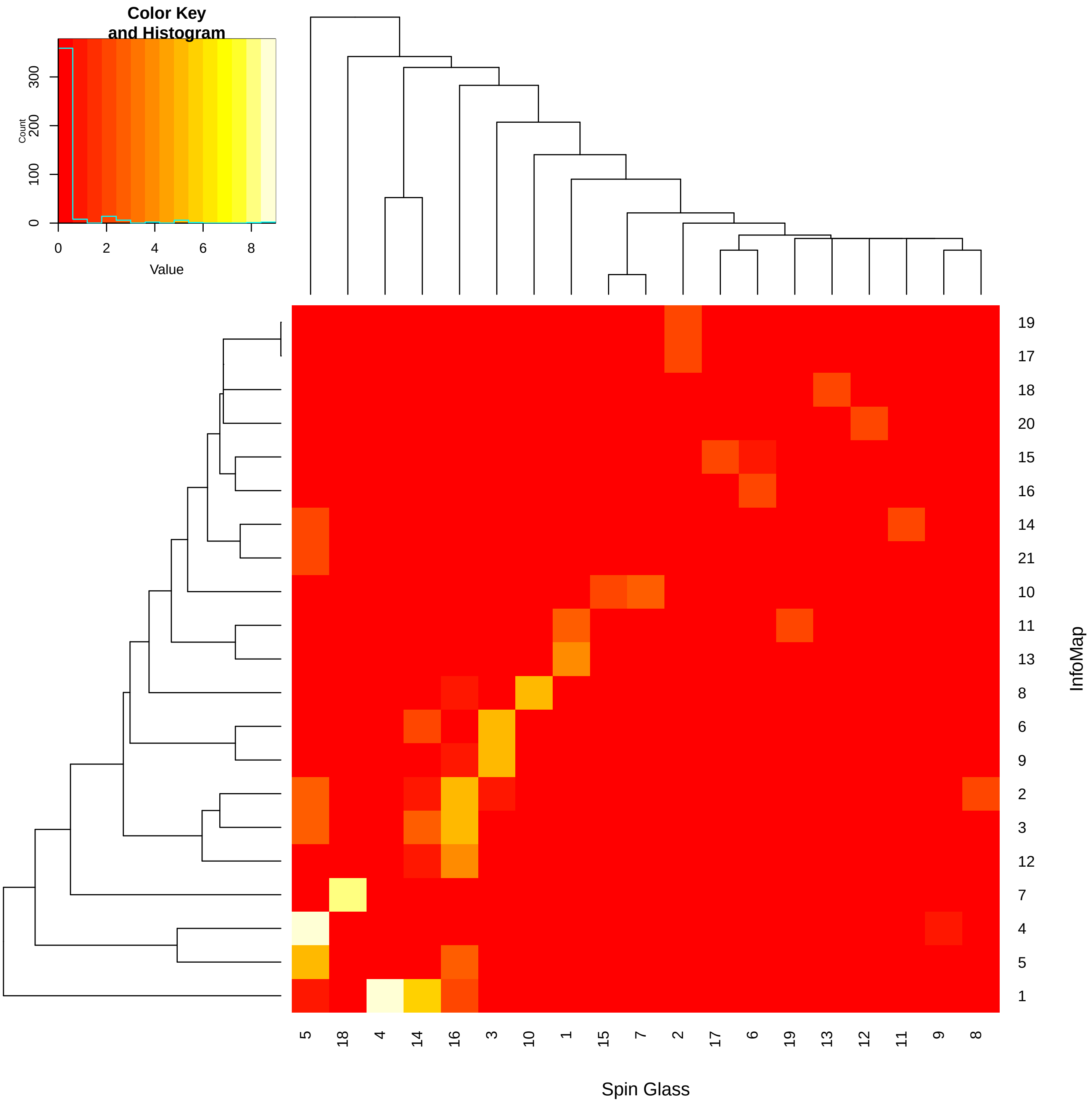

S27 Fig: Comparison of trait clusters produced by the Spin-Glass and InfoMap clustering algorithms. Cluster IDs for each method indicated on the x and y axes respectively. The color of each cell indicated the number of traits falling into that combination of cluster ID's. Note that for each cluster ID on the x-axis, there is a cluster id on the y-axis to which it roughly corresponds.

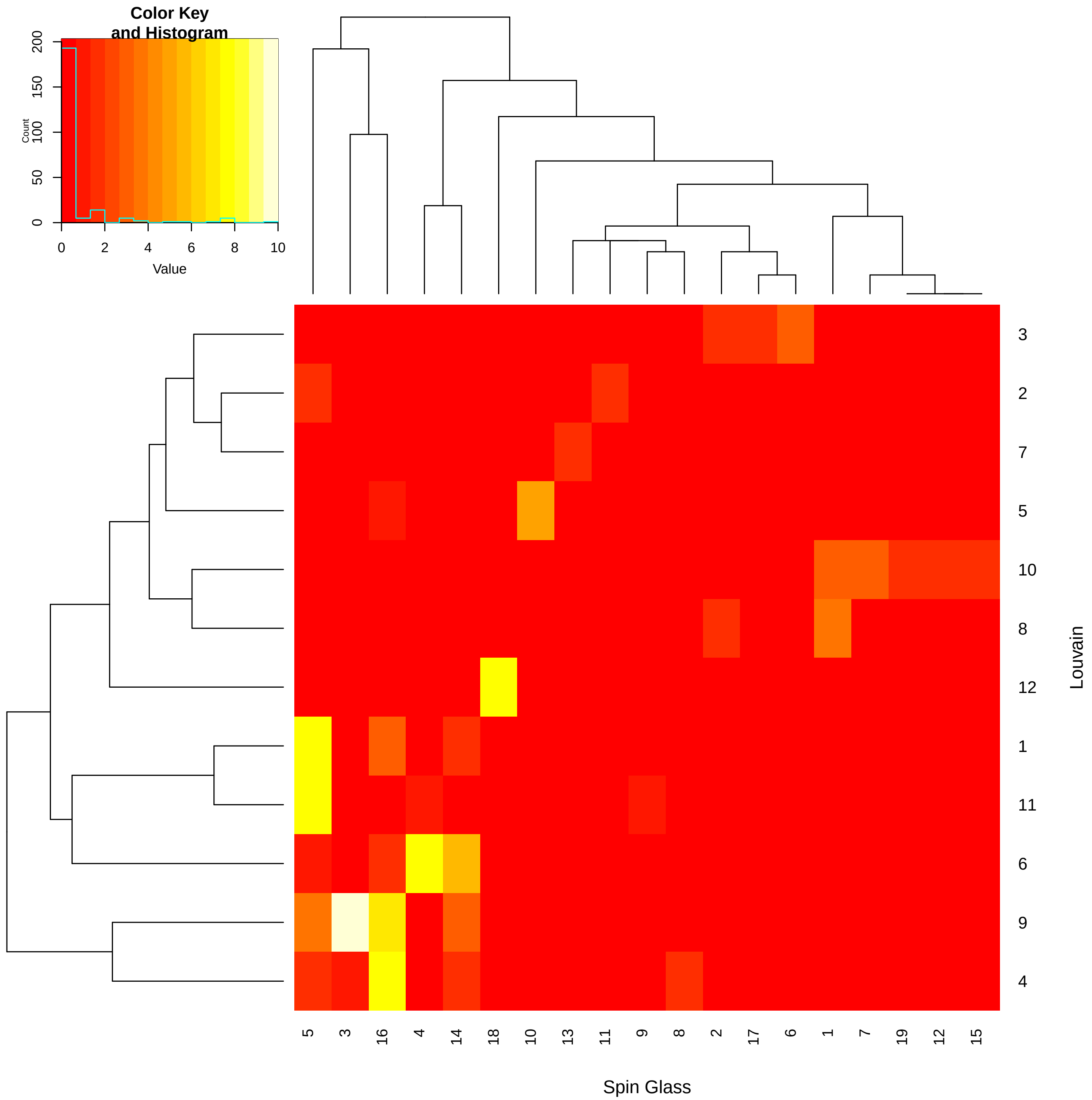

S28 Fig: Comparison of trait clusters produced by the Spin-Glass and Louvain clustering algorithms. Cluster IDs for each method indicated on the x and y axes respectively. The color of each cell indicated the number of traits falling into that combination of cluster ID's. Note that for each cluster ID on the x-axis, there is one or two cluster ids on the y-axis to which it roughly corresponds.

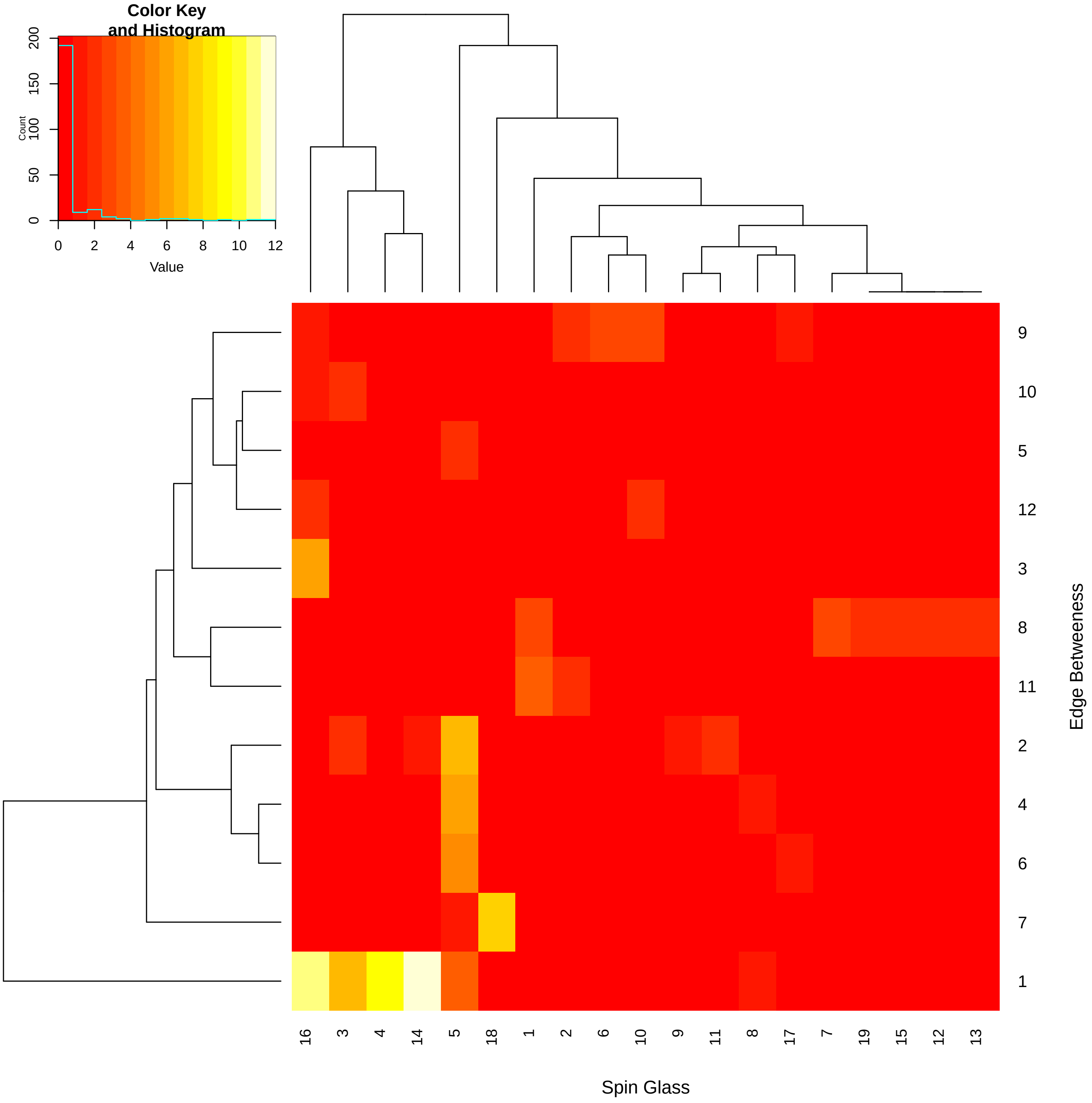

S29 Fig: Comparison of trait clusters produced by the Spin-Glass and Edge-Betweenness clustering algorithms. Cluster IDs for each method indicated on the x and y axes respectively. The color of each cell indicated the number of traits falling into that combination of cluster ID's. Note that for the most part these clustering algorithms do not agree.
